# Universal detection of phytoplasmas and *Xylella* spp. by TaqMan singleplex and multiplex real-time PCR with dual priming oligonucleotides

**DOI:** 10.1371/journal.pone.0185427

**Published:** 2017-09-28

**Authors:** Takao Ito, Koichi Suzaki

**Affiliations:** Division of Grape and Persimmon Research, Institute of Fruit Tree and Tea Science, National Agriculture and Food Research Organization (NARO), Higashihiroshima, Hiroshima, Japan; University of Helsinki, FINLAND

## Abstract

Phytoplasmas and *Xylella* spp. are bacteria that cause many economically important plant diseases worldwide. TaqMan probe-based quantitative real-time polymerase chain reaction (qPCR) assays have been utilized to universally detect phytoplasmas or *Xylella fastidiosa*. To develop a superior universal qPCR method, we used a dual priming oligonucleotide (DPO) with two annealing sites as a reverse primer to target the well-conserved bacterial 16S rDNA. The new qPCR assays universally detected various species of phytoplasmas and subspecies of *X*. *fastidiosa* as well as *Xylella taiwanensis*, and generally showed superior threshold cycle values when amplifying specific or non-specific products compared to current universal qPCR assays. The proposed qPCR assays were integrated to develop a multiplex qPCR assay that simultaneously detected phytoplasmas, *Xylella* spp., and an internal plant DNA positive control within 1 hour. This assay could detect a minimum of ten bacterial cells and was compatible with crude extractions used in the rapid screening of various plants. The amplicons were of sufficient lengths to be directly sequenced for preliminary identification, and the primers could be used in universal conventional PCR assays. Additionally, reverse DPO primers can be utilized to improve other probe-based qPCR assays.

## Introduction

‘*Candidatus* Phytoplasma’ (PP) and *Xylella* (XL) spp. are phloem- and xylem-limited fastidious bacteria, respectively. PP and XL have wide host ranges, with the ability to infect over 1,000 and 359 plants, respectively [1,2]. They cause economically important diseases in many plants, particularly woody plants such as citrus, grapevine, peach, and pear. For example, PP causes flavescence dorée in grapevine, citrus witches’ broom, stone fruit yellows, and pear decline. XL causes Pierce’s disease in grapevine, citrus variegated chlorosis, plum leaf scald, and pear leaf scorch [3,4]. These bacteria can severely affect host plants by reducing crop production and finally killing the plants. Additionally, they spread not only through vegetative propagation but also via insect transmission [1–4]. Infected plants are usually removed to eliminate sources of secondary infections. Outbreaks of new diseases caused by these bacteria have emerged and become worldwide threats. This includes olive quick decline syndrome in southern Italy, which is caused by *Xylella fastidiosa*. The disease spread from an initial estimation of 8,000 ha to 23,000 ha after just a few months [3]. XL includes *X*. *fastidiosa* and *Xylella taiwanensis*, which was until recently an unspecified subspecies of *X*. *fastidiosa* but is now classified as a new species [5,6]. *X*. *fastidiosa* includes at least three subspecies: *fastidiosa*, *multiplex*, and *pauca* [5]. PP consists of possibly 48 species, containing 33 groups having different 16S rDNA restriction fragment length polymorphism (16Sr) patterns [7]. In Japan, only nine PP species have been reported [8], and no XL has been found. Post-entry quarantine testing is the key to preventing the importation of plants infected with these regulated pathogens. However, they are difficult to identify based only on their symptoms, or to culture *in vitro* [1–4]. Several PCR-based molecular methods have been developed for their detection, and among these methods, TaqMan probe-based quantitative real-time PCR (qPCR) is the most sensitive [1,2]. Although qPCR requires special instruments (thermal cyclers with fluorescence detection), unlike conventional PCR (cPCR), it does not require laborious post-amplification processing [1,2]. Several universal qPCR assays to detect various species and subspecies of bacteria have already been used in plant quarantine screening. Standardized universal qPCR protocols for PP species [2] and *X*. *fastidiosa* subspecies [1] include those described by Christensen et al. (2004, 2013) [9,10] (qPCR-C2004/C2013) and Hodgetts et al. [11] (qPCR-Hd) as well as Francis et al. [12] (qPCR-F) and Harper et al. [13] (qPCR-Hp), respectively. Another qPCR protocol by Li et al. (qPCR-L) can also be used to detect various *X*. *fastidiosa* strains [14]. Each assay can be combined in a duplex qPCR to co-amplify an internal positive control from host plants [1,2,14]. However, qPCR to detect PP can result in false positives through amplification of non-specific products from closely related bacteria [2,9], while qPCR-F, qPCR-Hp, and qPCR-L do not target *X*. *taiwanensis* [13,14].

We investigated universal oligonucleotides (primers and probes) that specifically target PP and those that target *X*. *fastidiosa* as well as *X*. *taiwanensis*. Oligonucleotides for universal qPCR should anneal to specific and conserved nucleotide sequences in the target. Although a specific nucleotide sequence in a conserved gene is the best target, searching for these nucleotide sequences is difficult because conserved genes often have shared nucleotide sequences, such as bacterial 16S rDNA. This gene is often the most conserved and well-documented bacterial gene in sequence databases, which enables us to search for the most conserved sequences. To target 16S rDNA, we utilized a dual priming oligonucleotide (DPO). A DPO contains two separate annealing sites joined by a five deoxyinosine linker [15]. The linker prevents the formation of secondary structures, and effectively eliminates non-specific primer hybridization to targeted sequences [15]. Therefore, almost any sequence of interest can be used when designing DPO primers, resulting in a wide range of target sequences. DPO primers can generate consistently specific amplifications, even under non-optimal conditions and with complex PCR manipulations [15]. They have been successfully used in genotyping PCR, multiplex PCR, and SYBR Green-based qPCR assays to detect specific sequences [15,16]. To the best of our knowledge, this is the first report of the use of DPO primers in probe-based singleplex and multiplex qPCR assays. Here, we demonstrate that the proposed assays for detecting PP and XL have many advantages over the current standardized universal qPCR assays. Accordingly, DPO primers were effectively used as reverse primers to reduce non-specific amplifications.

## Materials and methods

### Samples

#### Preparation

Table 1 shows the samples utilized in this study, many of which were kindly provided by other researchers. Some XL strains, and most of the other bacteria were obtained from the American Type Culture Collection (ATCC, Manassas, VA, USA) and NARO Genebank (Ibaraki, Japan), respectively. Live pathogens from foreign countries were imported under special permission from the Minister of Agriculture, Forestry, and Fisheries, Japan. A grapevine infected with XL was obtained after needle infiltration by an XL (ATCC35879) suspension culture. A culture of *Escherichia coli* One Shot BL21 (DE3) pLysS chemically competent cells was purchased from Invitrogen (Carlsbad, CA, USA). The genomic DNA extract of *Acholeplasma laidlawii* was obtained from Minerva Biolabs GmbH (Berlin, Germany). Partial gene fragments of 400–480 base pairs were synthesized by Genewiz (South Plainfield, NJ, USA) or from gBlocks Gene Fragments (Integrated DNA Technologies, Coralville, IA, USA), using the sequence data of bacteria whose original DNAs were not obtained. Healthy plants were collected from our orchards, except for an olive tree purchased from a private nursery.

**Table 1:** Bacterial samples tested in this study.

Species	Description (Strain)	Material/ Source^c^	DNA concentration^d^
(‘*Candidatus* Phytoplasma’ spp.: PP)			
‘*Ca*. Pytoplasma asteris’ (16SrI) ^a^	Onion yellows (OY)	Garland chrysanthemum/ M. Tanaka (NARO, Japan)	1.5
	Paulownia witches' broom (PaWB)	DNA/ N. Nishimura (Koibuchi College of Agriculture and Nutrition, Japan)	0.5
	Porcelain vine witches’ broom (PvWB)	DNA/ H.-Y. Jung (Kyungpook national university, Korea)	2.5
	Rhus yellows (RhY)	DNA/ N. Nishimura	0.5
‘*Ca*. P. allocasuarinae’ (16SrXXXIII)	Allocasuarina yellows (AlloY)	Partial 16S rDNA of AY135523/ gBlocks (Integrated DNA Technologies, Coralville, IA, USA)	0.05
‘*Ca*. P. aurantifolia’ (16SrII)	chrysanthemum virescence (ChV)	DNA/ MAFF106058	0.5
	Faba bean phyllody (FBP)	DNA/ A. Bertaccini (University of Bologna, Italy)	5
‘*Ca*. P. australasiae’ (16SrII)	Pear decline Taiwan (PD-TWII)	DNA/ S.-C. Chang (Agricultural Research Institute, Taiwan)	0.5
	Tomato big bud (TBB)	DNA/ A. Bertaccini	5
‘*Ca*. P. australiense’ (16SrXII)	Australian grapevine yellows (AUSGY)	DNA/ A. Bertaccini	2.5
‘*Ca*. P. brasiliense’ (16SrXV)	Suriname virescence (SuV)	DNA/ A. Bertaccini	5
‘*Ca*. P. castaneae’ (16SrXIX)	Chestnut witches' broom (CnWB)	DNA/ H.-Y. Jung	5
‘*Ca*. P. cocostanzaniae’ (16SrIV)	Tanzanian coconut lethal decline (LDT)	Partial 16S rDNA of X80117/ gBlocks	0.05
‘*Ca*. P. convolvuli’ (16SrXII)	Convolvolus 57/11	DNA/ A. Bertaccini	5
‘*Ca*. P. costaricanum’ (16SrXXXI)	Soybean stunt (SoyST1c1)	Partial 16S rDNA of HQ225630/ gBlocks	0.05
‘*Ca*. P. fraxini’ (16SrVII)	Ash yellows (ASHY3)	DNA/ A. Bertaccini	5
‘*Ca*. P. japonicum’ (16SrXII)	Japanese hydrangea phyllody (JHP)	DNA/ S. Namba (University of Tokyo, Japan)	0.5
‘*Ca*. P. mali’ (16SrX)	Apple proliferation (AP-15)	DNA/ A. Bertaccini, E Seemüller (Julius Kuehn Institute, Germany)	2.5
	Apple proliferation (AT)	DNA/ E. Seemüller	3.5
	Apple proliferation (12/93)	DNA/ E. Seemüller	2.5
‘*Ca*. P. oryzae’ (16SrXI)	Rice yellow dwarf (RYD)	Rice/ M. Tanaka	3
‘*Ca*. P. phoenicium’ (16SrIX)	Naxos yellows (NaxY)	DNA/ A. Bertaccini	5
‘*Ca*. P. pruni’ (16SrIII)	Peach X-disease (GVX)	DNA/ E. Seemüller	2.5
	Gentian witches' broom (GW)	Garland chrysanthemum/ M. Tanaka	1
‘*Ca*. P. prunorum’ (16SrX)	Stone fruit yellows (ESFY)	DNA/ E. Seemüller	0.5
	Stone fruit yellows (GESFY)	DNA/ E. Seemüller	2.5
‘*Ca*. P. pyri’ (16SrX)	Pear decline (PD)	DNA/ E. Seemüller	0.5
	Peach yellow leaf roll (PYLR)	DNA/ E. Seemüller	2
‘*Ca*. P. rubi’ (16SrV)	Rubus stunt (RuS)	DNA/ A. Bertaccini	5
‘*Ca*. P. solani’ (16SrXII)	Bois noir (H618)	DNA/ A. Batlle (Institute for Research and Technology in Food and Agriculture, Spain)	22
	Bois noir (TF19C57)	Grapevine/ A. Batlle	38
‘*Ca*. P. trifolii’ (16SrVI)	Clover proliferation (CP-1)	DNA/ A. Bertaccini	5
‘*Ca*. P. ulmi’ (16SrV)	Elm witches’ broom (ULW)	DNA/ A. Bertaccini	5
‘*Ca*. P. vitis’ (16SrV)	Flavescence dorée (FD1)	DNA/ A. Batlle	5
	Flavescence dorée (FD2)	DNA/ A. Batlle	8
	Flavescence dorée (W1)	DNA/ B. Duduk (Institute of Pesticides and Environmental Protection, Serbia)	2.5
	Flavescence dorée (W2)	DNA/ B. Duduk	2.5
‘*Ca*. P. ziziphi’ (16SrV)	Jujube witches' broom (JWB)	DNA/ H.-Y. Jung	0.5
A potential new ‘*Ca*. Phytoplasma’ sp. (16SrXXV)	Weeping tea tree witches’ broom (WTWB)	Partial 16S rDNA of AF521672/ gBlocks	0.05
A potential new ‘*Ca*. Phytoplasma’ sp. (16SrXXVIII)^b^	Derbid phytoplasma (DP)	Partial 16S rDNA of AY744945/ gBlocks	0.05
(*Xylella* spp.: XL)			
*Xylella fastidiosa* subsp. *fastidiosa*	Almond leaf scorch (ALS-BC)	Partial 16S rDNA of AF536770/ gBlocks	0.05
	Elm leaf scorch (ELM-1)	Culture/ ATCC35873	19
	Grapevine Pierce’s disease (PCE-RR)	Grapevine/ ATCC35879	54
	Grapevine Pierce’s disease (PD5-2)	DNA/ W.-L. Deng (National Chung Hsing University, Taiwan)	1
	Oak leaf scorch (OAK)	Culture/ ATCC35874	14
	Oleander leaf scorch (Ann 1)	Culture/ ATCC700598	12
*X*. *fastidiosa* subsp. *multiplex*	Plum leaf scald (PLM G83)	Culture/ ATCC35871	10
*X*. *fastidiosa* subsp. *pauca*	Citrus variegated chlorosis (9a5c)	Partial 16S rDNA of AE003849/ gBlocks	0.05
	Coffee leaf scorch (CM1)	Partial 16S rDNA of AF536767/ gBlocks	0.05
	Olive quick decline syndrome (Xfp01)	DNA/ M. Saponari (Institute for Sustainable Plant Protection, Italy)	2
*Xylella taiwanensis*	Pear leaf scorch (PLS235)	DNA/ W.-L. Deng	1
(Other bacteria)			
*Acholeplasma brassicae*	O502	Partial 16S and 23S rDNA of FO681348/ Genewiz (South Plainfield, NJ, USA)	0.5
*Acholeplasma laidlawii*	PG8	DNA/ Minerva Biolabs GmbH (Berlin, Germany)	0.05
*Acholeplasma palmae*	J233	Partial 16S and 23S rDNA of FO681347/ Genewiz	0.5
*Arthrobacter* sp.	19B	Culture/ MAFF811001	250
*Bacillus subtilis*	BN-7901(TD)	Culture/ MAFF118079	250
*Burkholderia gladioli*	BRA 1	Culture/ MAFF302515	250
*Burkholderia gladioli* pv. *gladioli*	AZ 87108	Culture/ MAFF302543	250
*Clavibacter michiganensis* subsp. *michiganensis*	N6601	Culture/ MAFF301037	250
*Escherichia coli*	BL21(DE3) pLysS	Culture/ Invitrogen (Carlsbad, CA, USA)	250
*Paenibacillus* sp.	HS11R029	Culture/ MAFF550250	250
*Pseudomonas* fluorescens	Pseu1	Culture/ Y. Tomitaka (NARO, Japan)	250
	Pseu2	Culture/ Y. Tomitaka	250
*Ralstonia pickettii*	C-176	Culture/ Y. Tomitaka	250
*Ralstonia solanacearum*	K-1	Culture/ MAFF106603	250
*Rhizobium vitis*	GAg27	Culture/ MAFF663001	250
*Stenotrophomonas maltophilia*	K-14 (6I11)	Culture/ MAFF301689	250
*Xanthomonas albilineans*	T161	Culture/ MAFF311420	250
*Xanthomonas arboricola*	C1	Culture/ MAFF211922	250
*Xanthomonas campestris* pv. *citri*	N6101	Culture/ MAFF301077	250
*Xanthomonas oryzae* pv. *oryzae*	H-9101	Culture/ MAFF210548	250
*Xylophilus ampelinus*	BB-4	Culture/ A. Shinmura (Hokkaido Research Organization, Japan)	250

^a^ Groups of 16S rDNA restriction fragment length polymorphism (16Sr) patterns of phytoplasmas are indicated in parentheses.

^b^ Detected from an insect and recoded only as sequence data in 2004.

^c^ ATCC: American type culture collection (Manassas, VA, USA). MAFF: genetic resources of NARO Genebank (Ibaraki, Japan). The 400–480 base pairs of 16S and 23S rDNA were synthesized based on the nucleotide sequence data of the GenBank accession numbers, which are underlined.

^d^ ng/ reaction

Pure DNA was extracted from plant samples and bacterial cultures using Nucleon Phytopure (GE Healthcare, Buckinghamshire, UK), and suspended in TE buffer (10 mM Tris-HCl and 1 mM ethylenediaminetetraacetic acid, pH 8.0). The DNA concentrations obtained from the cultures of other bacteria were adjusted to the highest template concentration (250 ng) in order to evaluate non-specific amplifications in each assay. Partial gene fragments and DNA templates of the positive controls were diluted in TE buffer to obtain appropriate concentrations, which were quantified for PCR assays. As shown in Table 1, we did not equilibrate the DNA concentrations of all the templates obtained. However, the same DNA preparation was used to compare the performances of new assays to those of existing published assays.

#### Nucleotide sequencing analysis

A sequencing analysis was performed to confirm the partial 16S rDNA of the *X*. *taiwanensis* strain PLS235 that was detected using only the proposed qPCR assays. Amplicons were created using GoTaq Hot Start polymerase (Promega, Madison, WI, USA) and the primers fD1/rD1 (Table 2). They were directly sequenced in both directions on an ABI PRISM 3100 Genetic Analyzer (Applied Biosystems, Foster City, CA, USA) using a BigDye Terminator v3.1 cycle sequencing kit (Applied Biosystems) and the primers fD1/XylR2 (Table 2).

**Table 2:** Primers and probes used in this study.

Target	Gene	Primer/probe^a^	Sequence (5′-3′) and modification^b^	*Tm* (°C)^c^	Usage^d^	References
Pytoplasmas	16S rDNA	**UPH-F (F)**	CGTACGCAAGTATGAAACTTAAAGGA	58.9	qPCR, cPCR	[9,10]
		UPH-R (R)	TCTTCGAATTAAACAACATGATCCA	58.6	qPCR-C2004/C2013	[9,10]
		UPH-Pb (P)	FAM-TGACGGGACTCCGCACAAGCG-BHQ1	69.4	qPCR	[9,10]
		**UPH-P (P)**	FAM-TGACGGGACTCCGCACA-MGB	69	qPCR	[17]
		UPHr2 (R)	CGACAACCATGCACCACCTG	61.9	qPCR	This study
		**D-UPHr2 (R)**	CGACAACCATGCACCACCTGIIIIICTGATAACC	76.3	qPCR, cPCR	This study
	23S rDNA	JH-F 1 (F)	GGTCTCCGAATGGGAAAACC	59.2	qPCR-Hd	[11]
		JH-F all (F)	ATTTCCGAATGGGGCAACC	60.5	qPCR-Hd	[11]
		JH-R (R)	CTCGTCACTACTACCRGAATCGTTATTAC	58.7	qPCR-Hd	[11]
		JH-P uni (P)	FAM-AACTGAAATATCTAAGTAAC-MGB	65	qPCR-Hd	[11]
		OYrDr (R)	GACAAGATTTCTCGTGTCTCGC	57.7	Cloning	This study
*Xylella* spp.	16S rRNA processing protein	XF-F (F)	CACGGCTGGTAACGGAAGA	58.3	qPCR-Hp	[13]
		XF-R (R)	GGGTTGCGTGGTGAAATCAAG	61.2	qPCR-Hp	[13]
		XF-P (P)	FAM-TCGCATCCCGTGGCTCAGTCC-BHQ1	68.5	qPCR-Hp	[13]
	16S rDNA	XF16Sf (F)	CGGCAGCACGTTGGTAGTAA	58.5	qPCR-L	[14]
		XF16Sr (R)	CCGATGTATTCCTCACCCGTC	60.2	qPCR-L	[14]
		XF16Sp (P)	FAM-CATGGGTGGCGAGTGGC-BHQ1	60.6	qPCR-L	[14]
		**XrDf1 (F)**	GGCTCATCCAATCGCACAA	59.2	qPCR, cPCR	This study
		XrDr2 (R)	CGGACGGCAGCACAITGGTA	62.6	qPCR	This study
		**D-XrDr2 (R)**	CGGACGGCAGCACAITGGTAIIIIIACCATGG	80.3	qPCR, cPCR	This study
		D-XrDr9 (R)	CGGACGGCAGCACAITGGTAIIIIIACCATGGG	82.2	qPCR	This study
		XrD-Pf (P)	FAM-CCTAAGGTCCCCTGCTT-MGB	70	qPCR	This study
		**XrD-P (P)**	VIC-CCTAAGGTCCCCTGCTT-MGB	70	qPCR	This study
		XF1 (F)	CAGCACATTGGTAGTAATAC	44.3	cPCR	[18]
		XF6 (R)	ACTAGGTATTAACCAATTGC	45.3	cPCR	[18]
		X.fas-0838S (F)	GCAAATTGGCACTCAGTATCG	57	cPCR	[19]
		X.fas-1439A (R)	CTCCTCGCGGTTAAGCTAC	54	cPCR	[19]
		XylR2	CTACGCATTTCACTGCTACAC	52.5	Sequencing	This study
	RNA polymerase sigma factor	RST31 (F)	GCGTTAATTTTCGAAGTGATTCGATTGC	66.1	cPCR	[20]
		RST33 (R)	CACCATTCGTATCCCGGTG	58.2	cPCR	[20]
Bacteria	16S rDNA	FP1 (F)	AGAGTTTGATCCTGGCTCAGG	57.1	Cloning	[21]
		fD1 (F)	AGAGTTTGATCCTGGCTCAG	53.2	Sequencing	[22]
		rD1 (R)	AAGGAGGTGATCCAGCC	51.6	Sequencing	[22]
Plants	18S rDNA	**C18S-F2 (F)**	CAGCTCGCGTTGACTACGTC	58.3	qPCR	This study
		**D-C18Sr6 (R)**	GATCCGAACACTTCACCGGAIIIIICAATCGGTA	76.9	qPCR	This study
		**C18S-Pt (P)**	TAMRA-ACACACCGCCCGTCGCTCC-BHQ2a	67.5	qPCR	[9,10]

^a^ F: forward primer, R: reverse primer, P: probe. Those used in the final optimized TaqMan multiplex quantitative real-time PCR (qPCR) are indicated in bold.

^b^ FAM: 6-carboxyfluorescein, VIC: 2′-chloro-7′-phenyl-1,4-dichloro-6-carboxyfluorescein, TAMRA: 6-carboxytetramethylrhodamine, MGB: minor-groove-binding non-fluorescent quencher, BHQ: black hole quencher, I: inosine.

^c^ Melting temperature (*Tm*) values were predicted using the Primer Express software (Applied Biosystems, Foster City, CA, USA) or an oligonucleotide synthesis company (Fasmac Co., Ltd., Kanagawa, Japan; underlined), both of which use the nearest neighbor algorithm.

^d^ cPCR: conventional PCR. qPCR-C2004/C2013, qPCR-Hd, qPCR-Hp, and qPCR-L: qPCR assays of Christensen et al. [9,10], Hodgetts et al. [11], Harper et al. [13], and Li et al. [14], respectively.

#### Crude extractions from plants

For simple diagnosis in plant quarantines, we applied a crude extraction method. Leaves and petioles from grapevines infected with the PP strain TF19C57 and the XL strain PCE-RR (Table 1) were divided into samples of approximately 50 mg each. From each sample, pure and crude extracts were obtained. A pure extraction was performed using Nucleon Phytopure, which was finally suspended in 100 μl of TE buffer. A crude extraction was performed using a previously described method [23] with some modifications. A sample was mixed with 1 ml of extraction buffer (50 mM sodium citrate pH 8.3, 20 mM diethyldithiocarbamate, 2% polyvinylpyrrolidone K25, and 0.5% 2-mercaptoethanol) and then crushed with a metal bead using the Multi-beads shocker (Yasui kikai, Osaka, Japan) for 60 s at 2,500 rpm. The crude sap was centrifuged (9,000 ×*g* for 10 min at 4°C), and the supernatant was then transferred to a new tube. Then, 4 μl of the extract was added to 16 μl of 0.4% Triton X-100, heated at 75°C for 5 min, and then chilled on ice. Crude extracts were also obtained from leaf petioles of various healthy plants.

For isopropanol precipitations in order to concentrate the crude extracts, an equal volume of cold isopropanol was added to the supernatant of the crude sap and centrifuged (20,000 ×*g* for 5 min at 4°C). Pellets were dried without a final ethanol rinse and suspended in one-fifth volume of TE.

### Primers and probes

The primers and probes used in this study are listed in Table 2, and their targets are shown in S1 and S2 Figs. S1 Fig. includes all of the PP species reported by Zhao and Davis [7], and closely related bacteria in the class *Mollicutes*. S2 Fig. includes various XL strains and closely related bacteria of the order *Xanthomonadales*. S1 and S2 Figs show that the PP and XL strains used in this study (Table 1) fully represent the nucleotide sequence variation among the bacteria. We used the primer probe test tool of the Primer Express software v. 3.0.1 (Applied Biosystems) to manually identify primers and probes, and designed the DPO primers based on a previous study [15]. The reverse DPO primer D-UPHr2 can anneal its 5′- and 3′-segments to the conserved nucleotide sequences among the 16S rDNA of bacteria and the nine nucleotides conserved among the PP species, respectively. It ignores variable nucleotides through the use of the linker (S1 Fig). The reverse DPO primers D-XrDr2/D-XrDr9 can anneal to highly characteristic nucleotide sequences of *X*. *fastidiosa* [24], ignoring mismatches in some XL strains through the use of the linker (S2 Fig). The D-XrDr9 primer differs from the D-XrDr2 primer with the addition of a guanine at its 3′-terminus. The primers UPHr2 and XrDr2 represent the 5′-segments of the DPO primers D-UPHr2 and D-XrDr2/D-XrDr9, respectively. The probe UPH-P was modified from the UPH-Pb probe [17]. The probes XrD-Pf and XrD-P vary from one another only in their fluorescence reporters. The forward primer C18S-F2 and the reverse DPO primer D-C18Sr6 were selected to target the conserved 18S rDNA of plants using the probe reported by Christensen et al. [9,10]. The melting temperature values of the primers were calculated by Primer Express software or the oligonucleotide-synthesis company (Fasmac Co., Ltd., Kanagawa, Japan), using the nearest neighbor algorithm.

### qPCR assays

#### Singleplex and multiplex qPCR

All qPCR assays were performed using the same reaction mixture and parameters, except for the primer and probe concentrations of qPCR-C2004 [9] and qPCR-L [14], and the annealing/extension temperature of qPCR-Hp [13]. qPCR-C2004 used the same primers and probe as qPCR-C2013 [10] at 300 nM, 900 nM, and 200 nM for the forward primer, the reverse primer, and the probe, respectively [9]. In addition, qPCR-L used 240 nM of each primer and 120 nM of probe [14]. All of the other singleplex qPCR reactions used 300 nM of each primer and 100 nM of probe [10,11,13]. The multiplex qPCR used primers at 300 nM for PP and XL detection, and at 75 nM for plant detection, with probe concentrations of 50 nM, 100 nM, and 150 nM for PP, XL, and plant detection, respectively. All of the cPCR reactions were performed in a final volume of 10 μl containing 1X TaqMan Fast Advanced Master Mix (Applied Biosystems), primers, probes, and 0.5 μl of templates using the following fast-ramping parameters: uracil-N-glycosylase incubation for 2 min at 50°C and the first denaturation for 20 s at 95°C, followed by 50 cycles of denaturation for 1 s at 95°C, and primer annealing/extension for 20 s at 60°C (62°C for qPCR-Hp [13]) in the StepOnePlus system (Applied Biosystems). All samples were tested in triplicate. Cut-off threshold cycle (Ct) values were set at 45 cycles to assess PP and XL negative reactions in the multiplex qPCR only, and 40 cycles for plant in the multiplex qPCR and all singleplex qPCR assays [9−11,13,14]. Thermal cycling was performed for up to 50 cycles to evaluate each assay in detail. The protocol of the qPCR assays developed has been deposited in protocols.io (DOI: http://dx.doi.org/10.17504/protocols.io.jhjcj4n).

#### Standard curve analysis

Standard curves were constructed, and index values were calculated using the StepOnePlus software, with Ct values determined at < 40 in all of the triplicate samples, containing from 500 to 15.6 cells. The total DNA extracted from the *X*. *fastidiosa* strain ELM-1 (ATCC37853) culture was used as the standard for serial dilution of XL. The XL cell numbers (genome copies) were calculated using an estimated genome size of 2.5×10^6^ base pairs, and a previously described formula [13]. A plasmid containing PCR amplicons was used as a standard for the serial dilution of PP. Approximately 2,180 base pairs, including 16S and 23S rDNA, were PCR-amplified from the ‘*Ca*. Phytoplasma asteris’ strain OY (Table 1) using LA Taq (Takara, Shiga, Japan) with the primers FP1/OYrDr (Table 2). The amplicons were then inserted into the pCR2.1 vector using a TA cloning kit (Invitrogen). The inserted plasmid was prepared using a conventional alkaline lysis procedure, and further purified by polyethylene glycol precipitation. The plasmid numbers were calculated and then doubled to determine the cell numbers since PP has two rDNA operons in its genome [4]. Each XL and PP DNA sample was half-serially diluted from 500 to 3.9 cells with or without the DNA of an uninfected grapevine and another target, respectively. Also, for the multiplex qPCR, each sample was serially diluted 10-fold with TE (10 to 10^7^ cells per reaction) and tested in triplicate to determine the dynamic range. We tested 10 cells for a total of 21 reactions to estimate the limit of detection.

#### Data analysis

The statistical significance between two Ct values at the same threshold (0.02) was determined using the Student’s *t*-test. Ct values that were undetermined within 50 cycles were set as 50 for comparisons. Diagnostic sensitivity and specificity of qPCR assays were calculated from the reactions in each assay [25].

### cPCR assays

Universal cPCR assays using the new primers were also evaluated, and the assay for XL was compared to those previously reported [18−20]. All cPCR assays were performed using the protocol described by Loconsole et al. [26], with some modifications. Briefly, the cPCR reactions were performed in a final volume of 10 μl containing 1X GoTaq buffer (Promega) with 0.25 U of GoTaq Hot Start polymerase, 1.5 mM MgCl_2_, 160 μM dNTPs, 200 nM of each primer, and 0.5 μl of templates using the following cycling parameters: initial denaturation for 5 min at 95°C, then 35 or 43 cycles of denaturation for 30 s at 94°C, primer annealing for 30 s at 55°C (50°C for the XF1/XF6 primers), and primer extension for 40 s at 72°C, followed by a final extension for 7 min at 72°C in a GeneAmp 9700 thermal cycler (Applied Biosystems). Samples were considered positive when a DNA band of the expected size was clearly visualized after electrophoresis on a 1.5% agarose gel.

## Results

### Comparison of the singleplex qPCR assay using the reverse DPO primer with other universal qPCR assays

#### Phytoplasma detection

A new qPCR assay using the reverse DPO primer D-UPHr2 was performed using the UPH-Pb/UPH-F probe and primer [9,10]. The qPCR resulted in specific amplification products from all PP targets, and non-specific amplification products from two non-targets, *Acholeplasma brassicae* and *Acholeplasma palmae* (Table 3). Changing the primer from D-UPHr2 to UPHr2 decreased the Ct values (0.5 to 0.6) for the detection of the three targets as well as those for the non-specific amplification products (7.4 to 17.9) from three non-targets (Table 3). Changing the primer from D-UPHr2 to UPH-R reconstituted the probe/primer set for qPCR-C2013, which decreased the Ct values (0.3 to 3.9) when detecting some targets and increased the Ct values (> 9.4) for two other targets (Table 3). qPCR-C2013 also decreased the Ct values (2.5 to 13.7) of the non-specific amplification products from four non-targets (Table 3). qPCR-C2004 uses the same probe/primer set as qPCR-C2013 but at a higher concentration [9,10]. qPCR-C2004 decreased the Ct values of not only the specific amplification products (0.9 to 5.1), except for those of two targets, but also of the non-specific amplification products (1.5 to 19.2) (Table 3). qPCR-Hd increased the Ct values (1.8 to 17.5) to detect the targets, but failed to amplify products from a diluted sample of strain CnWB×10. This resulted in only one non-specific amplification product from *A*. *palmae* at a Ct value of 33.6 (Table 3). Consequently, the new assay showed either better sensitivity or specificity than qPCR-C2004/C2013 and qPCR-Hd (Table 3).

**Table 3:** Detection of phytoplasmas by TaqMan quantitative real-time PCR (qPCR) and conventional PCR (cPCR) with different probes and primers.

		qPCR with UPH-Pb/UPH-F and				qPCR with	qPCR-Hd	cPCR with
		D-UPHr2	UPHr2	UPH-R		UPH-P/UPH-F/D-UPHr2		UPH-F/D-UPHr2
Species	Strain			(qPCR-C2004^e^)	(qPCR-C2013)			
‘*Ca*. P. asteris’	OY	19.3 ± 0.1^c^	18.8 ± 0.1**/ -0.5^d^	17.6 ± 0.0**/ -1.7	19.5 ± 0.1	19.4 ± 0.1	21.7 ± 0.2**/ 2.4	+^g^
	PaWB	22.4 ± 0.0	nt	21.5 ± 0.0**/ -0.9	22.4 ± 0.0	23.6 ± 0.0**/ 1.2	26.4 ± 0.2**/ 3.9	+
	PvWB	20.9 ± 0.1	nt	19.6 ± 0.1**/ -1.3	20.8 ± 0.0	22.2 ± 0.2**/ 1.3	23.8 ± 0.1**/ 2.9	+
‘*Ca*. P. allocasuarinae’	Allo Y	12.2 ± 0.1	nt	7.1 ± 0.1**/ -5.1	8.3 ± 0.0**/ -3.9	11.5 ± 0.1*/ -0.7	nt	nt
‘*Ca*. P. aurantifolia’	FBP	15.8 ± 0.1	nt	14.2 ± 0.1**/ -1.7	15.3 ± 0.1**/ -0.5	15.9 ± 0.1	18.8 ± 0.2**/ 3.0	nt
‘*Ca*. P. brasiliense’	SuV	20.9 ± 0.0	nt	18.7 ± 0.0**/ -2.2	19.9 ± 0.0**/ -1.0	21.4 ± 0.0**/ 0.5	23.6 ± 0.1**/ 2.6	+
‘*Ca*. P. castaneae’	CnWB	26.7 ± 0.1	nt	23.8 ± 0.1**/ -2.9	26.7 ± 0.2	28.6 ± 0.0**/ 1.9	36.9 ± 0.6**/ 10.2	+
	CnWB×10^b^	31.2 ± 0.4	nt	29.0 ± 0.2**/ -2.2	30.4 ± 0.3	nt	(50.0 ± 0.0)**/ 18.8	nt
‘*Ca*. P. cocostanzaniae’	LDT	13.0 ± 0.0	nt	10.6 ± 0.1**/ -2.4	12.9 ± 0.1	12.0 ± 0.1**/ -1.0	nt	nt
‘*Ca*. P. costaricanum’	SoyST1c1	10.1 ± 0.1	nt	16.8 ± 0.0**/ 6.7	19.5 ± 0.1**/ 9.4	8.8 ± 0.1**/ -1.3	nt	nt
‘*Ca*. P. fraxini’	ASHY3	17.3 ± 0.1	nt	15.9 ± 0.0**/ -1.4	17.1 ± 0.1*/ -0.3	17.7 ± 0.0**/ 0.3	27.0 ± 0.2**/ 9.7	nt
‘*Ca*. P. mali’	AT	18.1 ± 0.0	nt	16.4 ± 0.1**/ -1.7	17.8 ± 0.0**/ -0.3	18.5 ± 0.1**/ 0.4	21.5 ± 0.0**/ 3.4	nt
‘*Ca*. P. oryzae’	RYD	19.7 ± 0.1	nt	18.8 ± 0.2*/ -1.0	19.9 ± 0.0	20.5 ± 0.0**/ 0.8	23.1 ± 0.3**/ 3.3	nt
‘*Ca*. P. phoenicium’	NaxY	22.5 ± 0.0	nt	20.5 ± 0.1**/ -2.0	22.7 ± 0.0	25.6 ± 0.0**/ 3.0	24.3 ± 0.0**/ 1.8	+
‘*Ca*. P. pruni’	GVX	15.8 ± 0.0	nt	14.6 ± 0.1**/ -1.3	15.9 ± 0.0	16.0 ± 0.1	33.4 ± 0.8**/ 17.5	nt
	GW	20.3 ± 0.0	nt	19.0 ± 0.1**/ -1.3	20.3 ± 0.1	20.9 ± 0.2*/ 0.6	31.4 ± 0.6**/ 11.1	+
‘*Ca*. P. prunorum’	GESFY	20.6 ± 0.1	nt	19.2 ± 0.2**/ -1.4	20.1 ± 0.1**/ -0.5	21.2 ± 0.1**/ 0.5	23.5 ± 0.2**/ 2.9	nt
‘*Ca*. P. pyri’	PD	20.9 ± 0.1	nt	19.9 ± 0.2**/ -1.0	21.1 ± 0.1	21.7 ± 0.1**/ 0.8	24.0 ± 0.3**/ 3.0	+
‘*Ca*. P. solani’	H618	33.1 ± 0.3	nt	30.0 ± 0.2**/ -3.1	32.0 ± 0.5	33.2 ± 0.2	37.9 ± 0.7**/ 4.8	+
	TF19C57	22.9 ± 0.2	22.4 ± 0.0*/ -0.5	21.1 ± 0.1**/ -1.9	23.3 ± 0.1	22.8 ± 0.1	26.8 ± 0.3**/ 3.9	nt
‘*Ca*. P. trifolii’	CP-1	23.8 ± 0.0	nt	22.2 ± 0.2**/ -1.6	23.5 ± 0.0**/ -0.3	23.8 ± 0.1	26.5 ± 0.2**/ 2.7	nt
‘*Ca*. P. vitis’	FD1	31.0 ± 0.1	30.4 ± 0.1*/ -0.6	28.3 ± 0.0**/ -2.7	30.7 ± 0.2	31.4 ± 0.1	33.7 ± 0.1**/ 2.7	+
	FD2	26.5 ± 0.1	nt	24.7 ± 0.1**/ -1.8	26.0 ± 0.2	26.7 ± 0.1	29.6 ± 0.1**/ 3.1	+
	W1	28.0 ± 0.1	nt	25.7 ± 0.0**/ -2.4	27.1 ± 0.1**/ -1.0	28.3 ± 0.1	30.8 ± 0.4**/ 2.7	+
‘*Ca*. Phytoplasma’ sp.	WTWB	10.0 ± 0.1	nt	7.4 ± 0.3**/ -2.7	8.1 ± 0.2**/ -1.9	9.6 ± 0.3	nt	nt
‘*Ca*. Phytoplasma’ sp.^a^	DP	13.0 ± 0.1	nt	25.6 ± 0.1**/ 12.6	28.7 ± 0.2**/ 15.7	(50.0 ± 0.0)**/ 37.0	nt	nt
(Other bacteria)								
*A*. *brassicae*	O502	12.5 ± 0.1	12.6 ± 0.1	11.0 ± 0.2**/ -1.5	12.8 ± 0.1	15.2 ± 0.0**/ 2.8	(50.0 ± 0.0^f^) **/ 37.5	+
*A*. *palmae*	J233	19.1 ± 0.3	11.8 ± 0.0**/ -7.4	10.5 ± 0.0**/ -8.6	12.7 ± 0.1**/ -6.4	22.2 ± 0.0**/ 2.8	33.6 ± 0.2**/ 14.5	+
*A*. *laidlawii*	PG8	(41.0 ± 1.4)	24.0 ± 0.1**/ -17.0	21.8 ± 0.3**/ -19.2	27.3 ± 0.1**/ -13.7	39.1 ± 0.3	(50.0 ± 0.0)**/ 9	(+)
*Arthrobacter* sp.	19B	(50.0 ± 0.0)	(50.0 ± 0.0)	(50.0 ± 0.0)	(50.0 ± 0.0)	(50.0 ± 0.0)	(50.0 ± 0.0)	–
*C*. *michiganensis*	N6601	(50.0 ± 0.0)	(50.0 ± 0.0)	(50.0 ± 0.0)	(50.0 ± 0.0)	(50.0 ± 0.0)	(50.0 ± 0.0)	–
*Paenibacillus* sp.	HS11R029	(42.9 ± 0.4)	25.0 ± 0.1**/ -17.9	34.5 ± 0.4**/ -8.3	(40.4 ± 0.2)**/ -2.5	(49.9 ± 0.1)**/ 7.1	(50.0 ± 0.0)**/ 7.1	–
*R*. *vitis*	GAg27	(50.0 ± 0.0)	nt	(41.9 ± 2.7)*/ -8.1	(50.0 ± 0.0)	(50.0 ± 0.0)	(50.0 ± 0.0)	–
*X*. *arboricola*	C1	(50.0 ± 0.0)	nt	(42.5 ± 0.4)**/ -7.5	(50.0 ± 0.0)	(50.0 ± 0.0)	(50.0 ± 0.0)	–
*X*. *ampelinus*	BB-4	(50.0 ± 0.0)	nt	35.7 ± 0.2**/ -14.3	(42.0 ± 0.3)**/ -8.0	(50.0 ± 0.0)	(50.0 ± 0.0)	–
(Healthy plants)								
*Vitis* sp. cv. Aurora black		(50.0 ± 0.0)	(50.0 ± 0.0)	38.6 ± 0.3**/ -11.4	(50.0 ± 0.0)	(50.0 ± 0.0)	(50.0 ± 0.0)	–
*Citrus jambhiri*		(50.0 ± 0.0)	nt	38.8 ± 0.3**/ -11.2	(45.5 ± 4.5)	(50.0 ± 0.0)	(50.0 ± 0.0)	–
Diagnostic sensitivity (%)		100	100	100	100	96	95.2	
Diagnostic specificity (%)		75.8	42.9	30.3	69.7	72.7	90.9	

^a^ Detected from an insect and recorded only as sequence data in 2004.

^b^ Ten-fold diluted samples of the CnWB strain.

^c^ Mean ± standard error (n = 3) of threshold cycle (Ct) values at an arbitrary threshold of 0.02. Undetermined Ct values within 50 cycles were temporally calculated as 50. nt: not tested. Mean values over 40 in parentheses were considered negatives.

^d^ A Student’s *t*-test was performed to compare the Ct values with those of the qPCR using UPH-Pb/UPH-F/D-UPHr2. The same DNA preparations were used, except when underlined, for the assay evaluations. Significant values are shown by asterisks: * *P* < 0.05, and ** *P* < 0.01. Significantly superior and inferior Ct values are highlighted in orange and gray, respectively, indicating differences in the values after slashes.

^e^ qPCR-C2004/C2013 and qPCR-Hd: qPCR assays of Christensen et al. [9,10], and Hodgetts et al. [11], respectively. qPCR-C2004 [9] uses the same probe and primers as qPCR-2013 [10] at a higher concentration.

^f^ A partial 23S rDNA fragment (indicated by the underline) was used as the template at the same concentration as the partial 16S rDNA fragment used in the other assays.

^g^ +: positive,–: negative, (+): positive signal diminished when the annealing temperature was set at 60°C, as in qPCR.

#### *Xylella* spp. detection

The proposed qPCR assay using the reverse DPO primer D-XrDr2 with the XrD-Pf/XrDf1 probe and primer detected all XL targets, and did not amplify any non-specific products from non-targets (Table 4). Changing the primer from D-XrDr2 to XrDr2 increased the Ct values (0.2 to 10.5) when detecting four targets, although it decreased the Ct values (6.6) when detecting the *X*. *taiwanensis* strain PLS235 (Table 4). The D-XrDr9 primer increased the Ct values (0.2 to 0.9) when detecting three targets and decreased these values (1.1 to 4.4) compared to the D-XrDr2 primer when detecting three other targets, including the PLS235 strain (Table 4). qPCR-Hp increased the Ct values (0.5 to 1.1) when detecting five targets, and failed to detect the PLS235 strain (Table 4). qPCR-L increased the Ct values (0.6 to 3.3) when detecting five targets, decreased the Ct values (1.5 to 6.9) when detecting two other targets, and failed to detect the PLS235 strain (Table 4). qPCR-L amplified non-specific products from four non-targets, among which *Xanthomonas albilineans* was especially clear at a Ct value of 15.8 (Table 4). Consequently, the proposed assays showed either better sensitivity or specificity than qPCR-Hp and qPCR-L (Table 4).

**Table 4:** Detection of *Xylella* by TaqMan quantitative real-time PCR (qPCR) and conventional PCR (cPCR) with different probes and primers.

		qPCR with XrD-Pf/XrDf1 and			qPCR-Hp^c^	qPCR-L	cPCR with			
Species	Strain	D-XrDr2	XrDr2	D-XrDr9			RST31/33	XF1/6	X.fas-0838S/1439A	XrDf1/D-XrDr2
*X*. *fastidiosa* subsp. *fastidiosa*	ALS-BC	10.6 ± 0.1^a^	21.1 ± 0.1**/ 10.5^b^	9.5 ± 0.0**/ -1.1	nt	13.9 ± 0.1**/ 3.3	nt/ nt	nt/ nt	nt/ nt	+/ nt
	ELM-1	12.7 ± 0.0	12.6 ± 0.1	12.9 ± 0.0**/ 0.2	13.8 ± 0.1**/ 1.1	13.5 ± 0.0**/ 0.8	+/ nt^d^	+/ nt	+/ nt	+/ nt
	PCE-RR	19.7 ± 0.1	19.4 ± 0.2	19.8 ± 0.1	19.8 ± 0.0	19.8 ± 0.0	+/ +	+/ +	+/ nt	+/ +
	PD5-2	23.8 ± 0.0	24.0 ± 0.1*/ 0.2	24.7 ± 0.0**/ 0.9	24.6 ± 0.1**/ 0.9	24.6 ± 0.0**/ 0.9	+/ +	+/ nt	+/ nt	+/ nt
	OAK	15.0 ± 0.1	nt	15.0 ± 0.1	15.5 ± 0.1*/ 0.5	15.4 ± 0.2	+/ nt	+/ nt	+/ nt	+/ nt
	Ann 1	13.9 ± 0.1	nt	14.0 ± 0.1	14.9 ± 0.1**/ 1.0	14.4 ± 0.1*/ 0.6	+/ nt	+/ nt	+/ nt	+/ nt
subsp. *multiplex*	PLM G83	13.7 ± 0.0	13.8 ± 0.1	14.2 ± 0.0**/ 0.5	14.6 ± 0.1**/ 1.0	14.2 ± 0.1**/ 0.6	+/ nt	+/ nt	+/ nt	+/ nt
subsp. *pauca*	9a5c	11.1 ± 0.0	11.4 ± 0.0**/ 0.3	11.3 ± 0.1	nt	9.6 ± 0.0**/ -1.5	nt/ nt	nt/ nt	nt/ nt	+/ nt
	CM1	17.3 ± 0.1	21.1 ± 0.1**/ 3.8	14.7 ± 0.0**/ -2.6	nt	10.4 ± 0.0**/ -6.9	nt/ nt	nt/ nt	nt/ nt	+/ nt
	Xfp01	21.0 ± 0.1	21.1 ± 0.1	20.9 ± 0.0	20.9 ± 0.2	20.8 ± 0.1	+/ +	+/ nt	+/ nt	+/ +
*X*. *taiwanensis*	PLS235	24.4 ± 0.1	17.8 ± 0.1**/ -6.6	20.0 ± 0.1**/ -4.4	(50 ± 0.0)**/ 25.6	(40.0 ± 0.4)**/ 15.7	–/ –	–/ –	+/ nt	+/ nt
(Other bacteria)										
*B*. *gladioli*	BRA 1	(50.0 ± 0.0)	(50.0 ± 0.0)	(50.0 ± 0.0)	(50.0 ± 0.0)	(50.0 ± 0.0)	nt/ nt	nt/ nt	–/ nt	nt/ –
*B*. *gladioli* pv. *gladioli*	AZ 87108	(48.7 ± 1.3)	nt	(50.0 ± 0.0)	(50.0 ± 0.0)	(50.0 ± 0.0)	nt/ nt	nt/ nt	–/ nt	nt/ –
*E*. *coli*	BL21(DE3) pLysS	(50.0 ± 0.0)	(50.0 ± 0.0)	(50.0 ± 0.0)	(50.0 ± 0.0)	(50.0 ± 0.0)	nt/ nt	nt/ nt	–/ nt	nt/ –
*R*. *pickettii*	C-176	(50.0 ± 0.0)	(50.0 ± 0.0)	(50.0 ± 0.0)	(50.0 ± 0.0)	(50.0 ± 0.0)	nt/ nt	nt/ nt	–/ nt	nt/ –
*S*. *maltophilia*	K-14 (6I11)	(50.0 ± 0.0)	(50.0 ± 0.0)	(50.0 ± 0.0)	(50.0 ± 0.0)	38.5 ± 0.7**/ -11.5	nt/ nt	nt/ nt	+/ nt	nt/ –
*X*. *albilineans*	T161	(50.0 ± 0.0)	(50.0 ± 0.0)	(50.0 ± 0.0)	(50.0 ± 0.0)	15.8 ± 0.0**/ -34.2	nt/ nt	nt /nt	+/ nt	nt/ –
*X*. *arboricola*	C1	(50.0 ± 0.0)	(50.0 ± 0.0)	(42.2 ± 7.8)	(50.0 ± 0.0)	37.6 ± 0.3**/ -12.4	–/ nt	–/ nt	+/ nt	nt/ –
*X*. *campestris* pv. *citri*	N6101	(50.0 ± 0.0)	(50.0 ± 0.0)	(50.0 ± 0.0)	(50.0 ± 0.0)	(46.1 ± 3.5)	–/ –	–/ –	+/ nt	nt/ –
*X*. *oryzae* pv. *oryzae*	H-9101	(50.0 ± 0.0)	(50.0 ± 0.0)	(50.0 ± 0.0)	(50.0 ± 0.0)	38.9 ± 1.3**/ -11.1	nt/ nt	nt/ nt	+/ nt	nt/ –
Diagnostic sensitivity (%)		100	100	100	87.5	97				
Diagnostic specificity (%)		100	100	96.3	100	55.6				

^a^ Mean ± standard error (n = 3) of the threshold cycle (Ct) values at an arbitrary threshold of 0.02. Undetermined Ct values within 50 cycles were temporarily calculated as 50; nt: not tested. Mean values over 40 in parentheses were considered negatives.

^b^ A Student’s *t*-test was performed to compare the Ct values with those of`the qPCR with XrD-Pf/XrDf1/D-XrDr2. The same DNA preparations were used for evaluating the assays. Significant values are shown as: * *P* < 0.05, ** *P* < 0.01. Significantly superior and inferior Ct values are highlighted in orange and gray, respectively, indicating differences in the values after slashes.

^c^ qPCR-Hp and qPCR-L: qPCR assays of Harper et al. [13] and Li et al. [14], respectively.

^d^ +: positive,–: negative at 35/ 43 cycles.

The nucleotide sequencing analysis showed that the PLS235 strain had a nucleotide sequence identical to those of the PL.788 and PE.PLS strains in the amplicon region (S2 Fig). The probe and primers of qPCR-L had more mismatches with those of the two XL strains they corresponded with those of some *Xanthomonas* spp., compared to those we designed (S2 Fig).

### Universal cPCR assays

cPCR using the primers UPH-F/D-UPHr2 with an annealing temperature of 55°C amplified products from all PP targets and *Acholeplasma* spp. without amplifying any non-specific products from the other bacteria after 43 cycles (Table 3). The detection results were consistent with those of qPCR using the primers and the UPH-Pb probe, except that cPCR detected *A*. *laidlawii*. The non-specific amplification of *A*. *laidlawii* was eliminated when the annealing temperature was set at 60°C, as in qPCR (Table 3).

cPCR with the new primers XrDf1/D-XrDr2 amplified products from all XL targets at 35 cycles and did not amplify products from the other bacteria, even at 43 cycles (Table 4). In contrast, the cPCR assays using the primers RST31/RST33 [20] and XF1/XF6 [18], amplified products from all of the tested *X*. *fastidiosa* strains at 35 cycles. However, they failed to amplify any products from the *X*. *taiwanensis* strain PLS235, even at 43 cycles (Table 4). cPCR with X.fas-0838S/X.fas-1439A [19] amplified a product from the PLS235 strain as well as non-specific products from five non-targets at 35 cycles (Table 4).

### Multiplex qPCR to detect phytoplasmas, *Xylella* spp., and an internal plant control

#### Probe and primer selection

To determine a probe for PP detection, we tested the UPH-P probe that was modified from the UPH-Pb probe (Table 2) for a multiplex RT-qPCR assay [17]. Although the UPH-P probe failed to detect the DP strain in the singleplex assay unlike the UPH-Pb probe (Table 3), the strain is recorded only as sequence data from one insect in 2004. qPCR using the probes UPH-P and UPH-Pb showed similar index values in the standard curve analysis (Table 5). UPH-P appeared to suppress non-specific amplification products from three non-targets at higher Ct values (2.8 to 7.1) than UPH-Pb (Table 3). Taking the degree of non-specific amplification into account, we selected the UPH-P probe for multiplex qPCR. To determine primers for XL detection, multiplex assays were performed using each of the primers D-XrDr2 and D-XrDr9. D-XrDr2 showed superior index values compared to D-XrDr9 in the standard curve analysis (Table 5). Therefore, we used the D-XrDr2 primer with the VIC-labeled XrD-P probe (Table 2).

**Table 5:** TaqMan quantitative real-time PCR (qPCR) assays to detect serially diluted ‘*Ca*. phtyoplasma asteris’ and *Xylella fastidiosa*.

Target^a^	Diluted with^b^	qPCR profile^c^	Ct values vs log DNA dilution^d^	*R*^*2*^	Efficiency (%)	7.8 cells	3.9 cells	Other target	Plant
‘*Ca*. P. asteris’	TE	UPH-F/UPH-R/UPH-Pb: qPCR-C2013	Y = -3.9 x + 43.3	0.97	80.2	35.8 ± 0.9^e^	**37.0 (2/3)**	na	na
	TE	JH-F1/JH-Fall/ JH-R/JH-P: qPCR-Hd	Y = -2.4 x + 38.9	0.88	159.8	36.7 ± 0.2	**38.0 (1/3)**	na	na
	TE	UPH-F/D-UPHr2/UPH-Pb	Y = -3.7 x + 43.2	0.94	86.4	35.9 ± 0.5	39.4 ± 3.6	na	na
	TE	UPH-F/D-UPHr2/UPH-P	Y = -3.8 x + 43.5	0.95	83.4	**35.2 (2/3)**	36.1 ± 0.3	na	na
	TE	Multiplex qPCR with D-XrDr2	Y = -3.4 x + 39.2	0.93	96.8	35.9 ± 1.4	**36.2 (2/3)**	50.0 ± 0.0	48.6 ± 0.6
	Plant1	qPCR-C2013	Y = -3.4 x + 38.4	0.95	95	35.9 ± 0.5	**36.2 (2/3)**	na	na
	Plant1	Multiplex qPCR with D-XrDr2	Y = -3.7 x + 39.7	0.97	87.7	**35.6 (2/3)**	36.5 ± 0.4	50.0 ± 0.0	23.6 ± 0.0
	Plant2/ *X*. *fastidiosa*	qPCR-C2013	Y = -3.7 x + 43.2	0.92	85.3	35.2 ± 0.3	**35.6 (2/3)**	na	na
	Plant2/ *X*. *fastidiosa*	Multiplex qPCR with D-XrDr2	Y = -4.7 x + 43.5	0.93	62.7	39.8 ± 0.3	**41.5 (1/3)**	18.5 ± 0.0	27.9 ± 1.0
	Plant2/ *X*. *fastidiosa*	Multiplex qPCR with D-XrDr9	Y = -6.9 x + 54.3	0.96	39.6	**44.5 (1/3)**	**46.2 (2/3)**	18.6 ± 0.0	27.4 ± 0.4
*X*. *fastidiosa*	TE	XF-F/XF-R/XF-P: qPCR-Hp	Y = -4.0 x + 43.1	0.91	78.5	**34.9 (1/3)**	34.8 ± 0.3	na	na
	TE	XfDf1/D-XrDr2/XrD-Pf	Y = -3.2 x + 36.5	0.96	105.3	**35.0 (2/3)**	**35.8 (1/3)**	na	na
	TE	Multiplex qPCR with D-XrDr2	Y = -3.3 x + 41.2	0.9	101	**35.7 (2/3)**	**36.8 (1/3)**	50.0 ± 0.0	48.9 ± 0.5
	TE	Multiplex qPCR with D-XrDr9	Y = -3.6 x + 42.1	0.92	88.6	35.8 ± 0.6	**37.2 (2/3)**	50.0 ± 0.0	43.2 ± 0.7
	Plant3	qPCR-Hp	Y = -3.5 x + 41.9	0.94	92.2	33.9 ± 0.2	**35.3 (2/3)**	na	na
	Plant3	Multiplex qPCR with D-XrDr2	Y = -3.4 x + 38.4	0.94	96.4	36.5 ± 0.7	35.8 ± 0.5	50.0 ± 0.0	24.4 ± 0.0
	Plant2/ ‘*Ca*. P. asteris’	qPCR-Hp	Y = -3.8 x + 42.2	0.93	83.3	34.4 ± 0.3	**34.6 (2/3)**	na	na
	Plant2/ ‘*Ca*. P. asteris’	Multiplex qPCR with D-XrDr2	Y = -4.0 x + 42.1	0.93	78.3	36.4 ± 0.3	36.2 ± 0.5	16.2 ± 0.0	26.0 ± 0.1
	Plant2/ ‘*Ca*. P. asteris’	Multiplex qPCR with D-XrDr9	Y = -4.3 x + 43.2	0.91	71.7	35.9 ± 0.2	**36.5 (2/3)**	16.2 ± 0.0	26.1 ± 0.1

^a^ Each target DNA of ‘*Ca*. P. asteris’ (the OY strain) and *X*. *fastidiosa* (the ELM-1 strain) was half-serially diluted from 500 to 3.9 bacterial cells (eight serial dilutions).

^b^ The target was diluted with TE buffer or healthy grapevine DNA of approximately 15 ng (Plant 1), 1.6 ng (Plant 2), or 11 ng (Plant 3) with or without another ‘*Ca*. P. asteris’ DNA target (7.9 × 10^6^ cells) or *X*. *fastidiosa* DNA (4.4 × 10^5^ cells) in each reaction.

^c^ Used primers and probes are indicated. qPCR-C2013, qPCR-Hd, and qPCR-Hp: qPCR assays of Christensen et al. [10], Hodgetts et al. [11], and Harper et al. [13], respectively. The other primers and probes used in the multiplex qPCR were UPH-F/D-UPHr2/UPH-P/XrDf1/XrD-P/C18S-F2/D-C18Sr6/C18S-Pt.

^d^ Standard curves calculated using the threshold cycle (Ct) values to detect 500 to 15.6 cells of the target (six serial dilution).

^e^ Mean ± standard error of Ct values at an arbitrary threshold of 0.02, n = 3 for 7.8 or 3.9 target cells. Bold font indicates that only one or two of the triplicates (shown in parentheses) were determined. n = 24 (triplicates × eight serial dilutions) for another target and plant, among which undetermined Ct values within 50 cycles were temporally calculated as 50. na: not analyzed.

#### Comparison of the multiplex qPCR assay to singleplex qPCR assays

The multiplex qPCR consistently detected 15.6 ‘*Ca*. P. asteris’ and *X*. *fastidiosa* cells from all of the triplicate reactions (Table 5). The assay resulted in some failures to detect one or two of the triplicates containing 7.8 or 3.9 cells. However, this was also observed in the singleplex qPCR assays (Table 5). The dynamic range for the multiplex qPCR to detect each of PP and XL was 10 to 10^7^ cells (S3 Fig), and its lower limit of detection was estimated as 10 cells from which 100% true positives were obtained from 21 reactions. The assay showed 100% diagnostic sensitivity and specificity to detect PP, XL, and plants, except for a false negative of PP from the DP strain, which the UPH-P probe failed to detect (Table 3), as well as PP false positives from three *Acholeplasma* spp. (Table 6). Amplification of PP generally showed higher Ct values (0.2 to 6.1) than qPCR-C2004/C2013 and lower Ct values (1.5 to 16.5) than qPCR-Hd (Table 6). The PP false positives from three *Acholeplasma* spp. were at higher Ct values (3.8 to 19.7) than those of qPCR-C2004/C2013 (Table 6). Amplification of XL generally showed higher Ct values (0.6 to 2.2) than those of qPCR-Hp, except for the *X*. *taiwanensis* strain PLS235, which qPCR-Hp failed to detect (Table 6).

**Table 6:** TaqMan multiplex qualitative real-time PCR (qPCR) to detect three targets and comparisons with other singleplex qPCR assays.

		Multiplex qPCR			qPCR for phytoplasmas			qPCR for *Xylella*
Species	Strain	Phytoplasma	*Xylella*	Plants	qPCR-C2004^d^	qPCR-C2013	qPCR-Hd	qPCR-Hp
‘*Ca*. P. asteris’	**OY**^b^	19.9 ± 0.0^c^	(50.0 ± 0.0)	24.8 ± 0.1	17.6 ± 0.0**/-2.2^e^	19.5 ± 0.1*/ -0.4	21.7 ± 0.2**/ 1.8	nt
	**PaWB**	23.9 ± 0.0	(50.0 ± 0.0)	24.5 ± 0.1	21.5 ± 0.0**/ -2.4	22.4 ± 0.0**/ -1.5	26.4 ± 0.2**/ 2.5	nt
	**PvWB**	21.8 ± 0.1	(50.0 ± 0.0)	22.1 ± 0.1	19.6 ± 0.1**/ -2.2	20.8 ± 0.0**/ -1.0	23.8 ± 0.1**/ 2.0	nt
	**RhY**	25.7 ± 0.1	(50.0 ± 0.0)	27.4 ± 0.1	23.1 ± 0.1**/ -2.6	nt	27.3 ± 0.2**/ 1.5	nt
‘*Ca*. P. allocasuarinae’	AlloY	13.1 ± 0.1	(50.0 ± 0.0)	(50.0 ± 0.0)	7.1 ± 0.1**/ -6.0	8.3 ± 0.0**/ -4.8	nt	nt
‘*Ca*. P. aurantifolia’	**ChV**	25.9 ± 0.1	(50.0 ± 0.0)	25.8 ± 0.0	23.3 ± 0.1**/ -2.7	nt	27.5 ± 0.1**/ 1.6	nt
	**FBP**	16.7 ± 0.0	(50.0 ± 0.0)	18.3 ± 0.1	14.2 ± 0.1**/ -2.5	15.3 ± 0.1**/ -1.4	18.8 ± 0.2**/ 2.1	nt
‘*Ca*. P. australasiae’	**PD-TWII**	22.4 ± 0.1	(50.0 ± 0.0)	23.4 ± 0.0	19.7 ± 0.0**/ -2.7	nt	24.4 ± 0.1**/ 2.1	nt
	**TBB**	17.9 ± 0.0	(50.0 ± 0.0)	19.1 ± 0.1	15.4 ± 0.1**/ -2.5	nt	19.6 ± 0.1**/ 1.7	nt
‘*Ca*. P. australiense’	**AUSGY**	32.5 ± 0.1	(50.0 ± 0.0)	25.0 ± 0.2	29.2 ± 0.2**/ -3.3	nt	35.4 ± 0.2**/ 2.9	nt
‘*Ca*. P. brasiliense’	**SuV**	22.1 ± 0.1	(50.0 ± 0.0)	19.7 ± 0.2	18.7 ± 0.0**/ -3.3	19.9 ± 0.0**/ -2.2	23.6 ± 0.1**/ 1.5	nt
‘*Ca*. P. castaneae’	**CnWB**	29.9 ± 0.1	(50.0 ± 0.0)	17.2 ± 0.2	23.8 ± 0.1**/ -6.1	26.7 ± 0.2**/ -3.2	36.9 ± 0.6**/ 6.9	nt
‘*Ca*. P. cocostanzaniae’	LDT	13.5 ± 0.2	(50.0 ± 0.0)	(50.0 ± 0.0)	10.6 ± 0.1**/ -2.9	12.9 ± 0.1*/ -0.6	nt	nt
‘*Ca*. P. costaricanum’	SoyST1c1	9.5 ± 0.0	(50.0 ± 0.0)	(50.0 ± 0.0)	16.8 ± 0.0**/ 7.3	19.5 ± 0.1**/ 10.0	nt	nt
‘*Ca*. P. convolvuli’	**Convolvolus 57/11**	24.8 ± 0.1	(50.0 ± 0.0)	20.0 ± 0.1	21.8 ± 0.0**/ -3.0	nt	27.6 ± 0.1**/ 2.8	nt
‘*Ca*. P. fraxini’	**ASHY3**	18.5 ± 0.0	(50.0 ± 0.0)	17.7 ± 0.2	15.9 ± 0.0**/ -2.6	17.1 ± 0.1**/ -1.4	27.0 ± 0.2**/ 8.5	nt
‘*Ca*. P. japonicum’	**JHP**	28.4 ± 0.2	(50.0 ± 0.0)	27.8 ± 0.1	26.4 ± 0.2**/ -1.9	nt	32.1 ± 0.3**/ 3.7	nt
‘*Ca*. P. mali’	**AP-15**	22.7 ± 0.0	(50.0 ± 0.0)	20.9 ± 0.1	20.0 ± 0.1**/ -2.7	nt	24.7 ± 0.1**/ 2.1	nt
	**AT**	18.8 ± 0.1	(50.0 ± 0.0)	17.3 ± 0.1	16.4 ± 0.1**/ -2.5	17.8 ± 0.0**/ -1.0	21.5 ± 0.0**/ 2.6	nt
	**12/93**	19.4 ± 0.2	(50.0 ± 0.0)	16.7 ± 0.3	17.4 ± 0.1**/ -2.0	nt	22.1 ± 0.1**/ 2.7	nt
‘*Ca*. P. oryzae’	**RYD**	20.9 ± 0.1	(50.0 ± 0.0)	23.2 ± 0.2	18.8 ± 0.2**/ -2.1	19.9 ± 0.0**/ -1.0	23.1 ± 0.3**/ 2.2	nt
‘*Ca*. P. phoenicium’	**NaxY**	26.6 ± 0.0	(50.0 ± 0.0)	18.4 ± 0.1	20.5 ± 0.1**/ -6.0	22.7 ± 0.0**/ -3.9	24.3 ± 0.0**/ -2.2	nt
‘*Ca*. P. pruni’	**GVX**	16.8 ± 0.0	(50.0 ± 0.0)	18.1 ± 0.1	14.6 ± 0.1**/ -2.3	15.9 ± 0.0**/ -0.9	33.4 ± 0.8**/ 16.5	nt
	**GW**	21.4 ± 0.1	(50.0 ± 0.0)	24.7 ± 0.2	19.0 ± 0.1**/ -2.4	20.3 ± 0.1**/ -1.1	31.4 ± 0.6**/ 10.0	nt
‘*Ca*. P. prunorum’	**GESFY**	21.6 ± 0.1	(50.0 ± 0.0)	19.9 ± 0.1	19.2 ± 0.2**/ -2.4	20.1 ± 0.1**/ -1.5	23.5 ± 0.2**/ 1.9	nt
	**ESFY**	18.8 ± 0.0	(50.0 ± 0.0)	17.5 ± 0.0	17.0 ± 0.4**/ -1.8	nt	20.9 ± 0.2**/ 2.1	nt
‘*Ca*. P. pyri’	**PD**	22.1 ± 0.0	(50.0 ± 0.0)	18.2 ± 0.1	19.9 ± 0.2**/ -2.2	21.1 ± 0.1**/ -1.0	24.0 ± 0.3**/ 1.8	nt
	**PYLR**	14.9 ± 0.0	(50.0 ± 0.0)	16.8 ± 0.2	13.4 ± 0.4*/ -1.6	nt	30.9 ± 0.0**/ 15.9	nt
‘*Ca*. P. rubi’	**RuS**	20.7 ± 0.0	(50.0 ± 0.0)	19.7 ± 0.2	17.9 ± 0.2**/ -2.8	nt	22.6 ± 0.0**/ 1.9	nt
‘*Ca*. P. solani’	**H618**	33.5 ± 0.1	(50.0 ± 0.0)	28.9 ± 0.1	30.0 ± 0.2**/ -3.6	32.0 ± 0.5*/ -1.5	37.9 ± 0.7**/ 4.4	nt
	**TF19C57**	23.5 ± 0.0	(50.0 ± 0.0)	20.2 ± 0.1	21.1 ± 0.1**/ -2.5	23.3 ± 0.1*/ -0.2	26.8 ± 0.3**/ 3.3	nt
‘*Ca*. P. trifolii’	**CP-1**	24.7 ± 0.1	(50.0 ± 0.0)	22.9 ± 0.1	22.2 ± 0.2**/ -2.5	23.5 ± 0.0**/ -1.2	26.5 ± 0.2**/ 1.8	nt
‘*Ca*. P. ulmi’	**ULW**	20.6 ± 0.1	(50.0 ± 0.0)	19.6 ± 0.1	17.5 ± 0.0**/ -3.1	nt	22.4 ± 0.1**/ 1.9	nt
‘*Ca*. P. vitis’	**FD1**	31.8 ± 0.1	(50.0 ± 0.0)	27.4 ± 1.6	28.3 ± 0.0**/ -3.5	30.7 ± 0.2**/ -1.1	33.7 ± 0.1**/ 1.9	nt
	**FD2**	27.2 ± 0.1	(50.0 ± 0.0)	23.4 ± 0.1	24.7 ± 0.1**/ -2.5	26.0 ± 0.2**/ -1.2	29.6 ± 0.1**/ 2.4	nt
	**W1**	28.7 ± 0.0	(50.0 ± 0.0)	22.8 ± 0.1	25.7 ± 0.0**/ -3.0	27.1 ± 0.1**/ -1.6	30.8 ± 0.4**/ 2.0	nt
	**W2**	28.8 ± 0.2	(50.0 ± 0.0)	23.3 ± 1.2	25.1 ± 0.1**/ -3.8	nt	30.5 ± 0.0**/ 1.7	nt
‘*Ca*. P. ziziphi’	**JWB**	25.1 ± 0.0	(50.0 ± 0.0)	25.3 ± 0.2	22.9 ± 0.0**/ -2.3	nt	27.9 ± 0.1**/ 2.7	nt
A ‘*Ca*. Phytoplasma’ sp.	WTWB	10.4 ± 0.2	(50.0 ± 0.0)	(48.6 ± 1.4)	7.4 ± 0.3**/ -3.0	8.1 ± 0.2**/ -2.3	nt	nt
A ‘*Ca*. Phytoplasma’ sp.^a^	DP	(50.0 ± 0.0)	(50.0 ± 0.0)	(47.6 ± 2.4)	25.6 ± 0.1**/ -24.4	28.7 ± 0.2**/ -21.3	nt	nt
*X*. *fastidiosa* subsp. *fastidiosa*	ALS-BC	(50.0 ± 0.0)	12.7 ± 0.0	(50.0 ± 0.0)	nt	nt	nt	nt
	ELM-1	(50.0 ± 0.0)	14.4 ± 0.2	(46.6 ± 1.8)	nt	nt	nt	13.8 ± 0.1*/ -0.6
	**PCE-RR**	(50.0 ± 0.0)	21.1 ± 0.0	20.5 ± 0.2	nt	nt	nt	19.8 ± 0.0**/ -1.3
	PD5-2	(50.0 ± 0.0)	26.1 ± 0.1	(50.0 ± 0.0)	nt	nt	nt	24.6 ± 0.1**/-1.5
	OAK	(50.0 ± 0.0)	16.4 ± 0.1	(49.2 ± 0.8)	nt	nt	nt	15.5 ± 0.1**/ -0.9
	Ann 1	(50.0 ± 0.0)	15.3 ± 0.2	(40.8 ± 0.5)	nt	nt	nt	14.9 ± 0.1
*X*. *fastidiosa* subsp. *multiplex*	PLM G83	(50.0 ± 0.0)	15.3 ± 0.1	(50.0 ± 0.0)	nt	nt	nt	14.6 ± 0.1*/ -0.7
*X*. *fastidiosa* subsp. *pauca*	9a5c	(50.0 ± 0.0)	12.7 ± 0.2	(50.0 ± 0.0)	nt	nt	nt	nt
	CM1	(50.0 ± 0.0)	19.2 ± 0.1	(48.1 ± 1.9)	nt	nt	nt	nt
	Xfp01	(50.0 ± 0.0)	23.0 ± 0.1	(42.9 ± 0.5)	nt	nt	nt	20.9 ± 0.2**/ -2.2
*X*. *taiwanensis*	PLS235	(50.0 ± 0.0)	26.4 ± 0.1	(45.6 ± 2.2)	tnt	n	nt	(50.0 ± 0.0)**/ 23.6
‘*Ca*. P. asteris’ + *X*. *fastidiosa*	**OY** + PD5-2	20.2 ± 0.2	26.1 ± 0.3	25.3 ± 0.3	17.8 ± 0.1**/ -2.4	nt	21.4 ± 0.3*/ 1.2	25.6 ± 0.2
(Other bacteria)								
*A*. *brassicae*	O502	16.6 ± 0.0	(50.0 ± 0.0)	(49.4 ± 0.6)	11.0 ± 0.2**/ -5.6	12.8 ± 0.1**/ -3.8	(50.0 ± 0.0 ^f^)**/ 33.4	nt
*A*. *palmae*	J233	24.0 ± 0.1	(50.0 ± 0.0)	(47.9 ± 2.1)	10.5 ± 0.0**/ -13.5	12.7 ± 0.1**/ -11.3	33.6 ± 0.2**/ 9.6	nt
*A*. *laidlawii*	PG8	41.5 ± 1.1	(50.0 ± 0.0)	(50.0 ± 0.0)	21.8 ± 0.3**/ -19.7	27.3 ± 0.1**/ -14.2	(50.0 ± 0.0)**/ 8.5	nt
*Arthrobacter* sp.	19B	(50.0 ± 0.0)	(50.0 ± 0.0)	(49.3 ± 0.7)	(50.0 ± 0.0)	(50.0 ± 0.0)	(50.0 ± 0.0)	nt
*B*. *subtilis*	BN-7901(TD)	(50.0 ± 0.0)	(50.0 ± 0.0)	(45.4 ± 2.3)	(41.8 ± 4.1)	(50.0 ± 0.0)	(50.0 ± 0.0)	nt
*B*. *gladioli*	BRA 1	(50.0 ± 0.0)	(50.0 ± 0.0)	(45.5 ± 2.4)	(49.8 ± 0.2)	nt	(50.0 ± 0.0)	(50.0 ± 0.0)
*B*. *gladioli* pv. *gladioli*	AZ 87108	(50.0 ± 0.0)	(50.0 ± 0.0)	(47.4 ± 2.6)	(50.0 ± 0.0)	nt	(50.0 ± 0.0)	(50.0 ± 0.0)
*C*. *michiganensis*	N6601	(50.0 ± 0.0)	(50.0 ± 0.0)	(45.2 ± 2.6)	(50.0 ± 0.0)	(50.0 ± 0.0)	(50.0 ± 0.0)	nt
*E*. *coli*	BL21(DE3) pLysS	(50.0 ± 0.0)	(50.0 ± 0.0)	(49.3 ± 0.7)	nt	nt	nt	(50.0 ± 0.0)
*Paenibacillus* sp.	HS11R029	(50.0 ± 0.0)	(50.0 ± 0.0)	(40.7 ± 0.1)	34.5 ± 0.4**/ -15.5	(40.4 ± 0.2)** / -9.6	(50.0 ± 0.0)	nt
*Pseudomonas* sp.	Pseu1	(50.0 ± 0.0)	(50.0 ± 0.0)	(45.5 ± 2.2)	(50.0 ± 0.0)	nt	(50.0 ± 0.0)	nt
	Pseu2	(50.0 ± 0.0)	(50.0 ± 0.0)	(45.4 ± 2.3)	(50.0 ± 0.0)	nt	(50.0 ± 0.0)	nt
*R*. *pickettii*	C-176	(50.0 ± 0.0)	(50.0 ± 0.0)	(45.5 ± 2.3)	(50.0 ± 0.0)	nt	(50.0 ± 0.0)	(50.0 ± 0.0)
*R*. *solanacearum*	K-1	(50.0 ± 0.0)	(50.0 ± 0.0)	(45.9 ± 2.1)	(50.0 ± 0.0)	nt	(50.0 ± 0.0)	(50.0 ± 0.0)
*R*. *vitis*	GAg27	(50.0 ± 0.0)	(50.0 ± 0.0)	(42.4 ± 0.9)	(41.9 ± 2.7)*/ -8.1	(50.0 ± 0.0)	(50.0 ± 0.0)	nt
*S*. *maltophilia*	K-14 (6I11)	(50.0 ± 0.0)	(50.0 ± 0.0)	(47.1 ± 1.5)	nt	nt	nt	(50.0 ± 0.0)
*X*. *albilineans*	T161	(50.0 ± 0.0)	(50.0 ± 0.0)	(45.9 ± 2.0)	nt	nt	nt	(50.0 ± 0.0)
*X*. *arboricola*	C1	(50.0 ± 0.0)	(50.0 ± 0.0)	(50.0 ± 0.0)	(42.5 ± 0.4)**/ -7.5	(50.0 ± 0.0)	(50.0 ± 0.0)	(50.0 ± 0.0)
*X*. *campestris* pv. *citri*	N6101	(50.0 ± 0.0)	(50.0 ± 0.0)	(50.0 ± 0.0)	(43.1 ± 1.3)**/ -6.9	nt	(50.0 ± 0.0)	(50.0 ± 0.0)
*X*. *oryzae* pv. *oryzae*	H-9101	(50.0 ± 0.0)	(50.0 ± 0.0)	(50.0 ± 0.0)	nt	nt	nt	(50.0 ± 0.0)
*X*. *ampelinus*	BB-4	(50.0 ± 0.0)	(50.0 ± 0.0)	(50.0 ± 0.0)	35.7±0.2**/ -14.3	(42.0 ± 0.3)**/ -8.0	(50.0 ± 0.0)	(50.0 ± 0.0)

^a^ Detected from an insect and recoded only as sequence data in 2004.

^b^ DNA extracts from plants are shown in bold.

^c^ Mean ± standard error (n = 3) of threshold cycle (Ct) values at an arbitrary threshold of 0.02. Undetermined Ct values within 50 cycles were temporally calculated as 50. nt: not tested. Mean values in parentheses were considered negatives: those over 45 for phytoplasma and *Xylella* detection in the multiplex qPCR, and those over 40 for the others.

^d^ qPCR-C2004/C2013, qPCR-Hd, and qPCR-Hp: qPCR assays of Christensen et al. [9,10], Hodgetts et al. [11], and Harper et al. [13], respectively. qPCR-C2004 [9] uses the same probe and primers as qPCR-2013 [10] at a higher concentration.

^e^ A Student’s *t*-test was performed to compare the Ct values with those of the multiplex qPCR. The same DNA preparations were used to evaluate the assays, except for those underlined, as seen in Tables 3 and 4. Significant values are shown as: * *P* < 0.05, ** *P* < 0.01. Significantly superior and inferior Ct values to detect pathogens are highlighted in orange and gray, respectively, indicating differences in the values after slashes.

^f^ A partial 23S rDNA fragment (indicated by the underline) was used as the template at the same concentration as the partial 16S rDNA fragment used in the other assays.

#### Detection from crude extracts

The multiplex qPCR amplified targets from not only pure extracts obtained using a commercial kit but also crude extracts obtained using the method described by Nakaune and Nakano [23]. However, the Ct values for the crude extracts were higher (6.9 to 8.5) than those for the pure extracts (Table 7). The values were lower when the crude extracts were concentrated with isopropanol. However, they were still higher (1.3 to 2.9) than those for the pure extracts (Table 7). The multiplex qPCR specifically amplified plant DNA from various healthy plants without any non-specific amplifications of PP or XL (Table 7).

**Table 7:** TaqMan multiplex real-time quantitative real-time PCR (qPCR) to detect three targets from various templates.

Samples	Extraction methods^a^	Multiplex qPCR		
		Phytoplasma	*Xylella*	Plants
The phytoplasma strain TF19C57	Pure extraction	23.5 ± 0.0^b^	(50.0 ± 0.0)	20.2 ± 0.1
	Crude extraction	32.0 ± 0.1**/ 8.5^c^	(50.0 ± 0.0)	27.1 ± 0.2**/ 6.9
	Crude extraction/ isopropanol	26.2 ± 0.2**/ 2.7	(50.0 ± 0.0)	21.5 ± 0.3**/ 1.3
The *X*. *fastidiosa* strain PCE-RR	Pure extraction	(50.0 ± 0.0)	18.7 ± 0.1	19.8 ± 0.1
	Crude extraction	(50.0 ± 0.0)	26.2 ± 0.0**/ 7.5	27.0 ± 0.2**/ 7.2
	Crude extraction/ isopropanol	(50.0 ± 0.0)	21.6 ± 0.1**/ 2.9	22.6 ± 0.1**/ 2.8
(Healthy plants)				
*Vitis* spp. cv. Aurora Black	Pure extraction	(50.0 ± 0.0)	(50.0 ± 0.0)	22.3 ± 0.1
*Vitis vinifera* cv. Ruby Okuyama	Pure extraction	(50.0 ± 0.0)	(50.0 ± 0.0)	23.1 ± 0.1
*Citrus jambhiri*	Pure extraction	(50.0 ± 0.0)	(50.0 ± 0.0)	21.7 ± 0.0
*Malus domestica*	Crude extraction	(50.0 ± 0.0)	(50.0 ± 0.0)	27.9 ± 0.2
*Actinidia deliciosa* cv. Hayward	Crude extraction	(50.0 ± 0.0)	(50.0 ± 0.0)	30.5 ± 0.1
*Olea europaea* cv. Manzanillo	Crude extraction	(50.0 ± 0.0)	(50.0 ± 0.0)	28.5 ± 0.1
*Ficus carica* cv. Masui Dauphine	Crude extraction	(50.0 ± 0.0)	(50.0 ± 0.0)	27.1 ± 0.2
*Prunus persica* cv. Akatsuki	Crude extraction	(50.0 ± 0.0)	(50.0 ± 0.0)	23.5 ± 0.0
*Pyrus pyrifolia*	Crude extraction	(50.0 ± 0.0)	(50.0 ± 0.0)	32.2 ± 0.1
*Hydrangea macrophylla*	Crude extraction	(50.0 ± 0.0)	(50.0 ± 0.0)	34.1 ± 0.1
TE buffer		(50.0 ± 0.0)	(50.0 ± 0.0)	(50.0 ± 0.0)

^a^ Pure extraction was obtained using a commercial kit. Crude extraction was obtained according to the method described by Nakaune and Nakano [23]. Additional isopropanol-precipitation was performed to concentrate crude extracts.

^b^ Mean ± standard error (n = 3) of threshold cycle (Ct) values at an arbitrary threshold of 0.02. Undetermined Ct values within 50 cycles were temporally calculated as 50. Mean values in parentheses were considered negatives: those over 45 for detection of phytoplasmas and *Xylella* spp. and those over 40 for detection of plants.

^c^ A Student’s *t*-test was performed to compare the Ct values with those from pure extracts in the same sample. Significantly inferior Ct values (** *P* < 0.01) are highlighted in gray, indicating differences in the values after slashes.

## Discussion

qPCR-C2004/C2013 targeting of the 16S rDNA of PP [9,10] can result in non-specific amplification from *Acholeplasma* spp. (Table 3), which are found in plant tissues and surfaces [27]. qPCR-L targeting of the 16S rDNA of *X*. *fastidiosa* [14] can result in clear non-specific amplification from *X*. *albilineans* (Table 4). The qPCR-C2004/C2013 and qPCR-L targets share similar nucleotide sequences with these bacteria (S1 and S2 Figs). We specifically targeted the 16S rDNA using a DPO primer that has two annealing sites. DPO primers showed much higher melting temperature values than probes and conventional primers (Table 2). Therefore, forward DPO primers seem to be inappropriate for probe-based qPCR assays because they can extend strands without cleaving probes that undergo insufficient annealing. In contrast, reverse DPO primers can extend specific antisense strands efficiently without interfering with the other oligonucleotides. A qPCR assay using a reverse DPO primer has four annealing sites (one for the conventional primer, one for the probe, and two for the DPO primer). Targeting multiple distinct nucleotide sequences can result in the selective and specific amplification of the targets [15]. In fact, the use of the DPO primer D-UPHr2 instead of the conventional primers UPH-R and UPHr2 decreased non-specific amplification (Table 3).

Diagnostic protocols for PP [2] recommend that PCR products should be sequenced if the outcome is critical. Sequencing of PCR amplicons is also recommended for subspecies determination of XL [1]. Very-short amplicons from qPCR-C2004/C2013, qPCR-Hp, and qPCR-L cannot be analyzed by direct sequencing [9,10,13,14]. We designed primers to amplify approximately 170 base pairs. Thus, they can be used in both qPCR and cPCR assays (Tables 3 and 4), and the amplicons can be directly sequenced. Primers suitable for both qPCR and cPCR assays have useful features compared to those suitable for only one application [28]. The use of cPCR assays to detect PP can provide advantages over nested PCR, which is the standard for PP diagnosis, but has cross-contamination risks because of the two PCR steps [2]. The PP and XL amplicons generally result in less than 97.5% sequence identity, which is the identification limit for a species [2,29], with other closely related bacteria. These amplicons demonstrated the highest (100%) nucleotide sequence identities among species or subspecies (S4 and S5 Figs). The data are sufficient for a preliminary identification of species or subspecies and to determine possible false positives from other closely-related bacteria (Table 3).

qPCR-Hd generally detected PP at higher Ct values than qPCR-C2004/C2013 (Table 3). Consequently, qPCR-Hd failed to detect positives from some diluted PP samples, such as CnWB×10, even at 50 cycles, while qPCR-C2004/C2013 detected clear positives (Table 3). qPCR-Hd targets 23S rDNA, which can be more variable among PP species than 16S rDNA [11]. Therefore, it was unclear whether qPCR-Hd could detect PP species whose 16S rDNA sequences were only recorded in databases (Table 3). Fewer non-specific amplification products (Table 3) and amplicons long enough to be directly sequenced offer advantages of qPCR-Hd [11] over qPCR-C2004/C2013. The proposed qPCR assay using the D-UPHr2 primer has the most advantages. This new assay generates long amplicons and showed superior Ct values when detecting specific amplification products than qPCR-Hd. In addition, it had fewer non-specific amplification products than qPCR-C2004/C2013 (Table 3). Both UPH-Pb and UPH-P probes work well in the new qPCR assay, although the UPH-P generally resulted in higher Ct values when detecting both specific and non-specific products than UPH-Pb, and failed to detect the DP strain (Table 3). The UPH-P probe can be blocked by a 12-nucleotide insertion into the DP strain (S1 Fig). To our knowledge, the DP strain and its characteristic insertion have not been detected in any plants and other PP strains, respectively. Therefore, we do not believe that this failure is problematic for current plant quarantine screening.

We did not perform comparison tests with qPCR-F for XL detection because it fails to detect some *X*. *fastidiosa* strains [1,13,14]. Neither qPCR-F, qPCR-Hp, nor qPCR-L can detect *X*. *taiwanensis* [13] (Table 4). Because *X*. *taiwanensis* has been detected from pear leaf scorch disease in Taiwan [6], its detection is especially important for Asian plant quarantines. We designed primers and a probe that targeted the conserved 16S rDNA sequences among XL (S2 Fig). The new qPCR assay, incorporating the new probe and primers, successfully detected not only all of the tested *X*. *fastidiosa* strains but also *X*. *taiwanensis* (Table 4). Among the new reverse primers, the D-XrDr2 primer generally showed superior Ct values when detecting *X*. *fastidiosa* strains than the XrDr2 primer (Table 4), and performed better than the D-XrDr9 primer in the multiplex assays (Table 5). The D-XrDr9 primer contains three serial guanines at the 3′-terminus, which represents an additional guanine compared to the D-XrDr2 primer (Table 2). It may interfere with probes and conventional forward primers mixed in the multiplex qPCR. Consequently, we utilized the D-XrDr2 primer for the superior universal detection of XL. qPCR using the D-XrDr2 primer generally shows superior Ct values for specific and non-specific amplification products compared with qPCR-Hp and qPCR-L (Table 4). cPCR using the D-XrDr2 primer detects XL more accurately than cPCR assays using the other universal primers (Table 4).

The multiplex qPCR assay successfully detected PP, XL, and plants, although a reduction in the probe concentration (from 100 nM to 50 nM) and a change in the reporter (from FAM to VIC) could increase Ct values more than in the singleplex qPCR when detecting PP and XL, respectively (Tables 2–4 and 6). In the standard curve analysis (Table 5), the multiplex qPCR assay did not show inferior index values compared to those of qPCR-C2013, qPCR-Hd, and qPCR-Hp. The multiplex qPCR assay co-amplified the three targets with inferior PCR efficiencies (< 78.3%), during which the massive amplification of another target (> 4.4 × 10^5^ cells) could interfere with the amplification of a low amount of the target (< 500 cells) (Table 5). We set 45 cycles as the cut-off value to judge PP and XL negatives in the multiplex qPCR, while the cut-off value was set at 40 cycles for plant detection in the multiplex qPCR and singleplex qPCR assays [9–11,13]. The multiplex qPCR assay did not amplify any non-specific products from PP and XL, even at 50 cycles, or from other bacteria, except for *Acholeplasma* spp. However, it detected non-specific products from plants at Ct values of 40–50 (Table 6). Although the assay amplified a non-specific product at a Ct value of 41.5 from *A*. *laidlawii*, qPCR-C2004/C2013 amplified clearer non-specific products at Ct values of < 27.3 (Table 6). A fast ramping program can finish even 50 cycles within an hour. The multiplex qPCR detected a minimum of 10 ‘*Ca*. P. asteris’ and *X*. *fastidiosa* cells, and sometimes obtained positive amplifications from 7.8 and 3.9 cells, similar to singleplex qPCR assays (Table 5). The direct sequencing of some amplicons from the qPCR assays confirmed the specificity of the amplification (data not shown). The co-amplification of the internal plant control in the multiplex qPCR reveals PCR errors. The assay detected plant DNA from various herbaceous, woody, and monocotyledonous plants (Tables 1, 6 and 7). The multiplex qPCR amplifies less non-specific products than qPCR-C2004/C2013, amplifies products from almost all of the tested PP strains at superior Ct values than qPCR-Hd, and amplifies both *X*. *fastidiosa* and *X*. *taiwanensis* (Table 6). In practice, even qPCR-C2004 and qPCR-Hd, which produce different Ct values (Table 6), are similar in terms of sensitivity and specificity when amplifying ‘*Ca*. P. mali’, ‘*Ca*. P. prunorum’, and ‘*Ca*. P. pyri’ [2,25]. Although the multiplex qPCR amplified the PP and XL strains at higher Ct values than qPCR-C2004/C2013 and qPCR-Hp, the multiplex qPCR is as sensitive at 45 cycles as the singleplex qPCR assays is within 40 cycles (Tables 5 and 6).

The multiplex qPCR assay detected three targets from crude extracts using the simple method described by Nakaune and Nakano [23] (Table 7). This crude extraction method has been used routinely in the screening of grapevines infected with viruses and viroids [23]. Ct values from crude extracts were reduced when the method was combined with simple isopropanol precipitation (Table 7). Many samples can be crudely extracted and isopropanol-precipitated within an hour. The crude extracts can be used in a time-, cost-, and labor-saving qPCR screening assay.

## Conclusions

The new singleplex and multiplex qPCR assays demonstrated here have advantages over the current standardized universal qPCR assays used to detect PP and XL. In particular, the multiplex qPCR will be helpful for plant quarantine screening in countries that restrict the importation of PP- and XL-infected plants. Use of the DPO primer as a reverse primer has contributed to the superior specificity and accuracy of the qPCR assays, and this concept can be applied to improve other probe-based qPCR assays. The amplicons are of adequate lengths to be directly sequenced for preliminary identification, and the primers can also be used in universal cPCR assays.

## Supporting information

S1 Fig Sequence alignment of partial 16S rDNA genes of ‘*Candidatus* phytoplasma’ species and other bacteria. Bacterial names are followed by GenBank accession numbers and strain names are shown in parentheses. The ‘*Ca*. Phytoplasma’ species used in this study and other bacteria in the class *Mollicutes* are highlighted in gray and black, respectively. Nucleotides matching the top two lines and blanks are shown as dots and bars, respectively. The DP strain, which was detected from an insect and recorded only as sequence data in 2004, is indicated by an asterisk. The positions of the primers and probes used in this study are boxed.
(PDF)

S2 Fig Sequence alignments of the partial 16S rDNA genes of *Xylella* strains and other bacteria. Bacterial names are followed by GenBank accession numbers and strain names are shown in parentheses. The *X*. *fastidiosa* strains used in this study and other bacteria in the order *Xanthomonadales* are highlighted in gray and black, respectively. The identical nucleotide sequence as the *X*. *taiwanensis* strain PLS235 used in this study is indicated by an asterisk. Nucleotides matching the top two lines and blank spaces are represented by dots and bars, respectively. The positions of the primers and probes used in this study are boxed. Primer and probe positions used in qPCR-L [14] are underlined.
(PDF)

S3 Fig **Dynamic range of TaqMan multiplex quantitative real-time PCR to detect (A) ‘*Candidatus* Phytoplasma asteris’ and (B) *Xylella fastidiosa*, which were reduced to 10 cells with 7-log dilutions**.
(PDF)

S4 Fig Nucleotide sequence identities among 16S rDNA regions located between the UPH-F and D-UPHr2 primers. Bacterial names are followed by GenBank accession numbers. Each of the P1–36 (phytoplasmas, boxed) and O1–7 (other bacteria in the class *Mollicutes*) has a unique nucleotide sequence. Only identity levels near the 97.5% species identification threshold are shown to the first decimal place. Identity levels greater than 97.5% are highlighted in gray.
(PDF)

S5 Fig Nucleotide sequence identities among 16S rDNA regions located between the primers XrDf1 and D-XrDr2. Bacterial names are followed by GenBank accession numbers. Each of the X1–5 (*Xylella*, boxed) and O1–4 (Other bacteria in the order *Xanthomonadales*) has a unique nucleotide sequence. Only identity levels near the 97.5% species identification threshold are shown to the first decimal place. Identity levels greater than 97.5% are highlighted in gray.
(PDF)

S1 Dataset Nucleotide sequence files (*.seq) of 16S rDNA regions located between the UPH-F and D-UPHr2 primers of phytoplasmas and other bacteria. (ZIP)

S2 Dataset Nucleotide sequence files (*.seq) of 16S rDNA regions located between the XrDf1 and D-XrDr2 primers of *Xylella* spp. and other bacteria. (ZIP)
