# Supplementary material for: Universal detection of phytoplasmas and Xylella spp. by TaqMan singleplex and multiplex real-time PCR with dual priming oligonucleotides
Source: PLoS One. 2017 Sep 28;12(9):e0185427. doi: 10.1371/journal.pone.0185427 (PMC5619750; doi:10.1371/journal.pone.0185427)
Supplement: S1 Fig — Bacterial names are followed by GenBank accession numbers and strain names are shown in parentheses. The ‘Ca. Phytoplasma’ species used in this study and other bacteria in the class Mollicutes are highlighted in gray and black, respectively. Nucleotides matching the top two lines and blanks are shown as dots and bars, respectively. The DP strain, which was detected from an insect and recorded only as sequence data in 2004, is indicated by an asterisk. The positions of the primers and probes used in this study are boxed. (PDF) [file pone.0185427.s001.pdf]

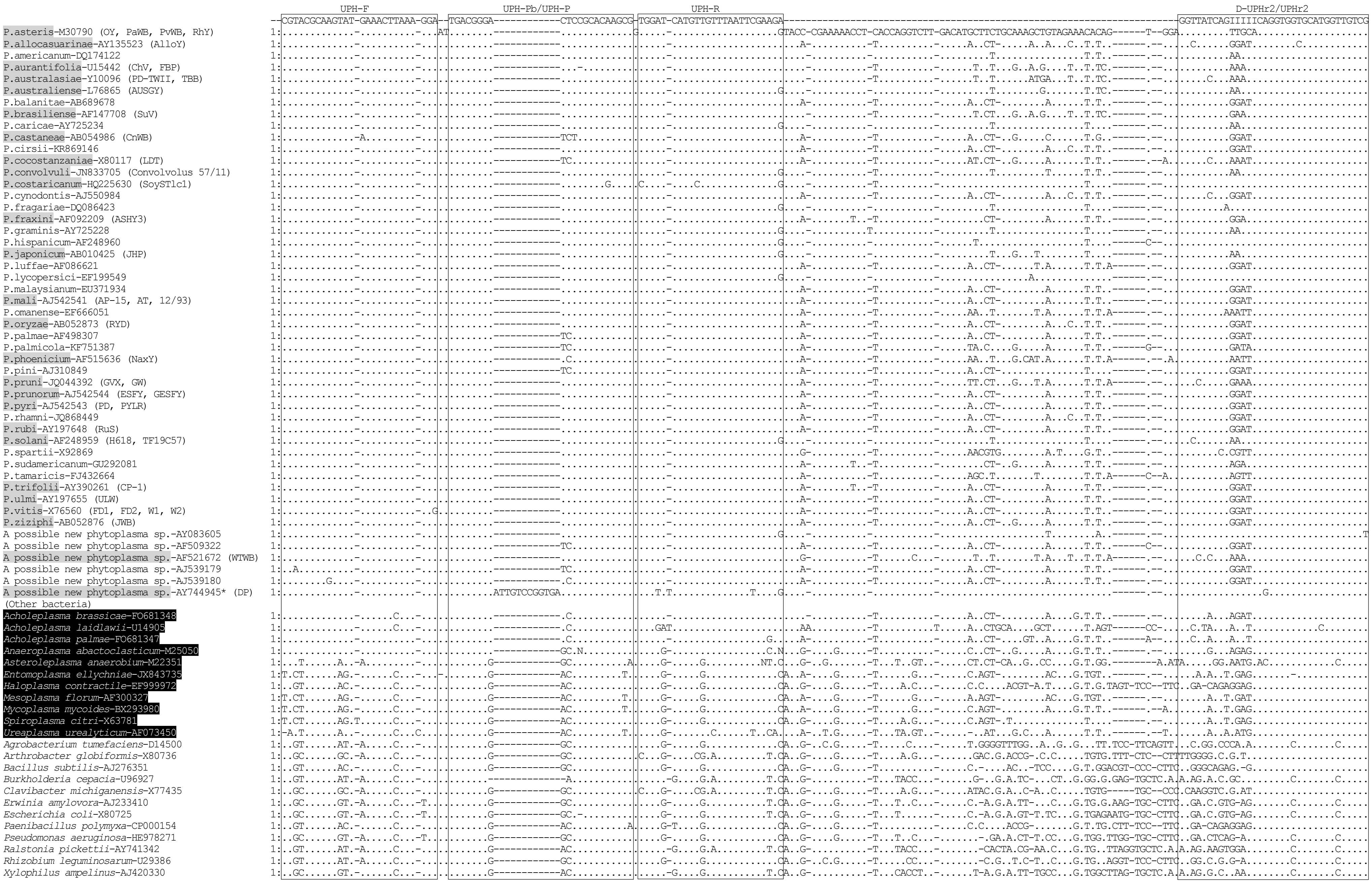
UPH-F
UPH-Pb/UPH-P
UPH-R
D-UPHr2/UPHr2
--CGTACGCAAGTAT-GAAACTTAAA-GGA--TGACGGGA-------------CTCCGCACAAGCG-TGGAT-CATGTTGTTTAATTCGAAGA
GTACC-CGAAAAACCT-CACCAGGTCTT-GACATGCTTCTGCAAAGCTGTAGAAACACAG------T--GGA
GGTTATCAGIIIIICAGGTGGTGCATGGTTGTCG
P.asteris-M30790 (OY, PaWB, PvWB, RhY)
P.allocasuarinae-AY135523 (AlloY)
P.americanum-DQ174122
P.aurantifolia-U15442 (ChV, FBP)
P.australasiae-Y10096 (PD-TWII, TBB)
P.australiense-L76865 (AUSGY)
P.balanitae-AB689678
P.brasiliense-AF147708 (SuV)
P.caricae-AY725234
P.castaneae-AB054986 (CnWB)
P.cirsii-KR869146
P.cocostanzaniae-X80117 (LDT)
P.convolvuli-JN833705 (Convolvulus 57/11)
P.costaricanum-HQ225630 (SoySTlc1)
P.cynodontis-AJ550984
P.fragariae-DQ086423
P.fraxini-AF092209 (ASHY3)
P.graminis-AY725228
P.hispanicum-AF248960
P.japonicum-AB010425 (JHP)
P.luffae-AF086621
P.lycopersici-EF199549
P.malaysianum-EU371934
P.mali-AJ542541 (AP-15, AT, 12/93)
P.omanense-EF666051
P.oryzae-AB052873 (RYD)
P.palmae-AF498307
P.palmicola-KF751387
P.phoenicium-AF515636 (NaxY)
P.pini-AJ310849
P.pruni-JQ044392 (GVX, GW)
P.prunorum-AJ542544 (ESFY, GESFY)
P.pyri-AJ542543 (PD, PYLR)
P.rhamni-JQ868449
P.rubi-AY197648 (RuS)
P.solani-AF248959 (H618, TF19C57)
P.spartii-X92869
P.sudamericanum-GU292081
P.tamaricis-FJ432664
P.trifolii-AY390261 (CP-1)
P.ulmi-AY197655 (ULW)
P.vitis-X76560 (FD1, FD2, W1, W2)
P.ziziphi-AB052876 (JWB)
A possible new phytoplasma sp.-AY083605
A possible new phytoplasma sp.-AF509322
A possible new phytoplasma sp.-AF521672 (WTWB)
A possible new phytoplasma sp.-AJ539179
A possible new phytoplasma sp.-AJ539180
A possible new phytoplasma sp.-AY744945* (DP)
(Other bacteria)
Acholeplasma brassicae-FO681348
Acholeplasma laidlawii-U14905
Acholeplasma palmae-FO681347
Anaeroplasma abactoclasticum-M25050
Asteroleplasma anaerobium-M22351
Entomoplasma ellychniae-JX843735
Haloplasma contractile-EF999972
Mesoplasma florum-AF300327
Mycoplasma mycoides-BX293980
Spiroplasma citri-X63781
Ureaplasma urealyticum-AF073450
Agrobacterium tumefaciens-D14500
Arthrobacter globiformis-X80736
Bacillus subtilis-AJ276351
Burkholderia cepacia-U96927
Clavibacter michiganensis-X77435
Erwinia amylovora-AJ233410
Escherichia coli-X80725
Paenibacillus polymyxa-CP000154
Pseudomonas aeruginosa-HE978271
Ralstonia pickettii-AY741342
Rhizobium leguminosarum-U29386
Xylophilus ampelinus-AJ420330
