# Supplementary material for: Universal detection of phytoplasmas and Xylella spp. by TaqMan singleplex and multiplex real-time PCR with dual priming oligonucleotides
Source: PLoS One. 2017 Sep 28;12(9):e0185427. doi: 10.1371/journal.pone.0185427 (PMC5619750; doi:10.1371/journal.pone.0185427)
Supplement: S2 Fig — Bacterial names are followed by GenBank accession numbers and strain names are shown in parentheses. The X. fastidiosa strains used in this study and other bacteria in the order Xanthomonadales are highlighted in gray and black, respectively. The identical nucleotide sequence as the X. taiwanensis strain PLS235 used in this study is indicated by an asterisk. Nucleotides matching the top two lines and blank spaces are represented by dots and bars, respectively. The positions of the primers and probes used in this study are boxed. Primer and probe positions used in qPCR-L [14] are underlined. (PDF) [file pone.0185427.s002.pdf]

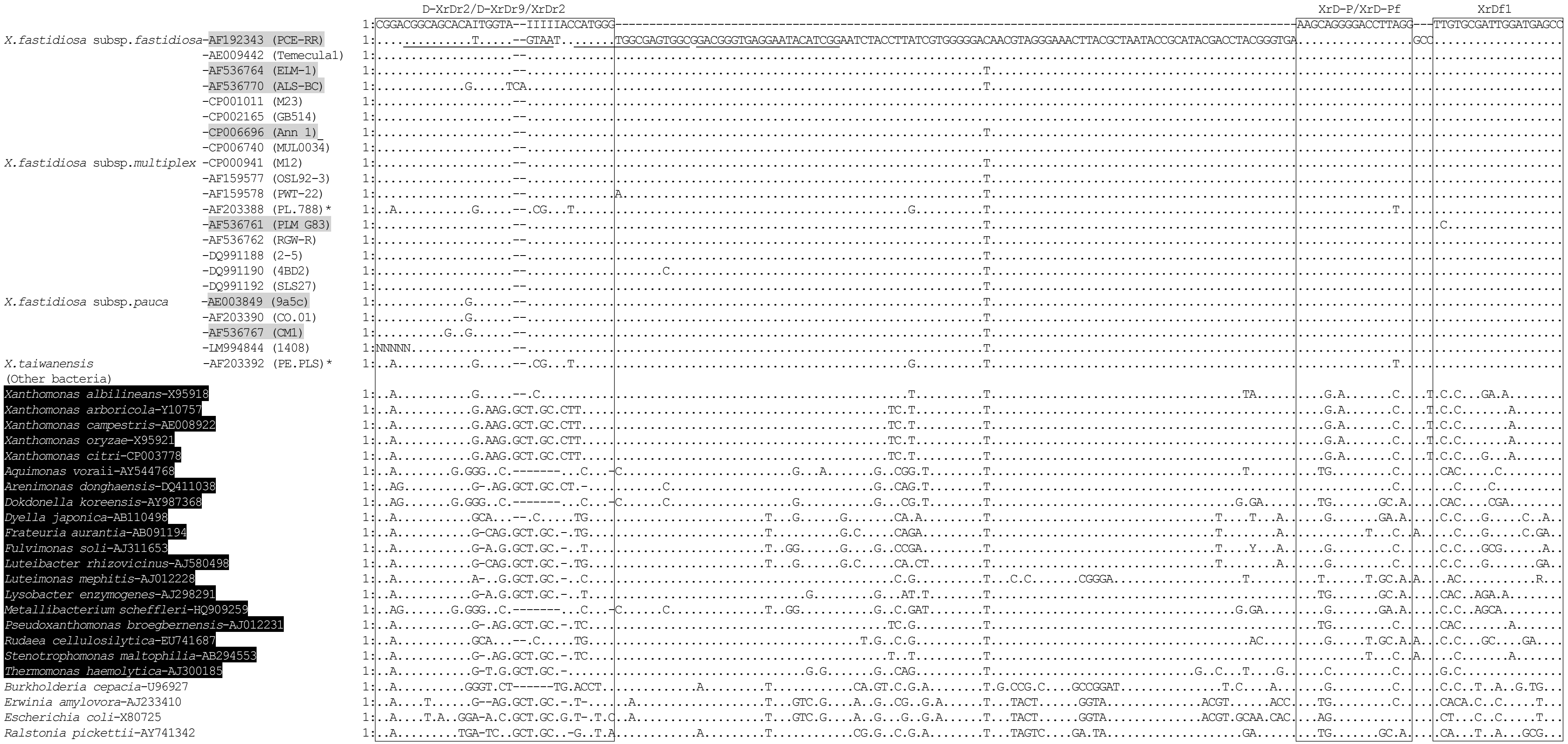
D-XrDr2/D-XrDr9/XrDr2
XrD-P/XrD-Pf
XrDf1
X.fastidiosa subsp.fastidiosa-AF192343 (PCE-RR)
-AE009442 (Temecula1)
-AF536764 (ELM-1)
-AF536770 (ALS-BC)
-CP001011 (M23)
-CP002165 (GB514)
-CP006696 (Ann 1)
-CP006740 (MUL0034)
X.fastidiosa subsp.multiplex -CP000941 (M12)
-AF159577 (OSL92-3)
-AF159578 (PWT-22)
-AF203388 (PL.788)*
-AF536761 (PLM G83)
-AF536762 (RGW-R)
-DQ991188 (2-5)
-DQ991190 (4BD2)
-DQ991192 (SLS27)
X.fastidiosa subsp.pauca -AE003849 (9a5c)
-AF203390 (CO.01)
-AF536767 (CM1)
-LM994844 (1408)
X.taiwanensis -AF203392 (PE.PLS)*
(Other bacteria)
Xanthomonas albilineans-X95918
Xanthomonas arboricola-Y10757
Xanthomonas campestris-AE008922
Xanthomonas oryzae-X95921
Xanthomonas citri-CP003778
Aquimonas voraii-AY544768
Arenimonas donghaensis-DQ411038
Dokdonella koreensis-AY987368
Dyella japonica-AB110498
Frateuria aurantia-AB091194
Fulvimonas soli-AJ311653
Luteibacter rhizovicinus-AJ580498
Luteimonas mephitis-AJ012228
Lysobacter enzymogenes-AJ298291
Metallibacterium scheffleri-HQ909259
Pseudoxanthomonas broegbernensis-AJ012231
Rudaea cellulosilytica-EU741687
Stenotrophomonas maltophilia-AB294553
Thermomonas haemolytica-AJ300185
Burkholderia cepacia-U96927
Erwinia amylovora-AJ233410
Escherichia coli-X80725
Ralstonia pickettii-AY741342
