# Supplementary figures and images for: Universal detection of phytoplasmas and Xylella spp. by TaqMan singleplex and multiplex real-time PCR with dual priming oligonucleotides
Source: PLoS One. 2017 Sep 28;12(9):e0185427. doi: 10.1371/journal.pone.0185427 (PMC5619750; doi:10.1371/journal.pone.0185427)

(A)

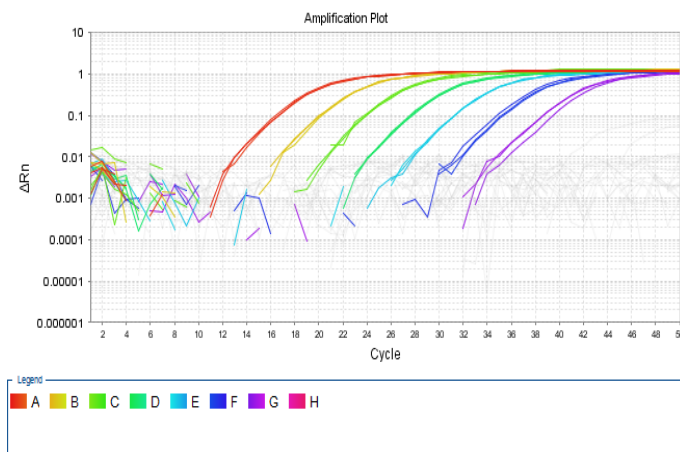

Standard Curve

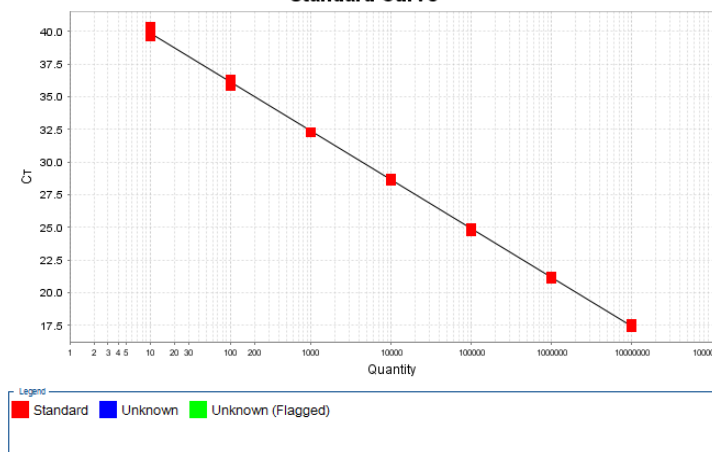

Slope:-3.734, Y-inter:43.576,  $R^2$ :0.999, Eff(%):85.284

(B)

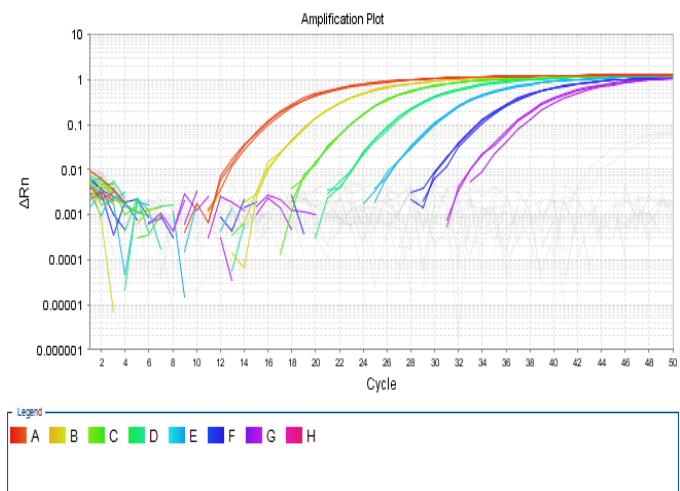

Standard Curve

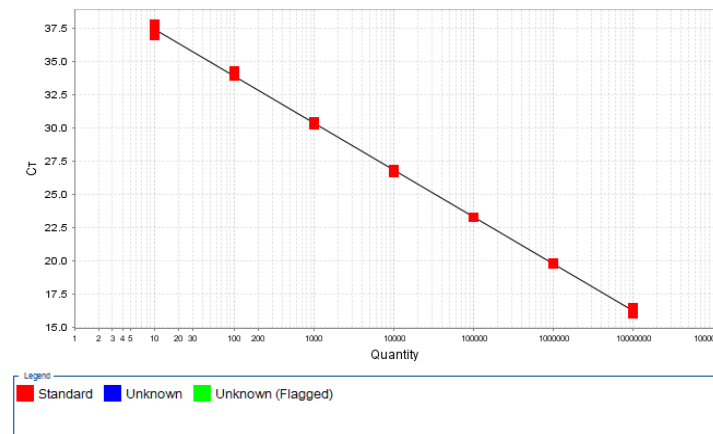

Slope:-3.536, Y-inter:40.94,  $R^2$ :0.999, Eff(%):91.799

Supplement: S3 Fig — Dynamic range of TaqMan multiplex quantitative real-time PCR to detect (A) ‘Candidatus Phytoplasma asteris’ and (B) Xylella fastidiosa, which were reduced to 10 cells with 7-log dilutions. (PDF) [file pone.0185427.s003.pdf]
